# The Role of Polydimethylsiloxane in Suppressing the Evolution of Lipid Oxidation Products in Thermo-Oxidised Sunflower Oil: Influence of Stirring Processes

**DOI:** 10.3389/fnut.2021.721736

**Published:** 2021-08-10

**Authors:** Gilbert Ampem, Adam Le Gresley, Martin Grootveld, Declan P. Naughton

**Affiliations:** ^1^Department of Chemistry and Pharmaceutical Sciences, Science, Engineering, and Computing Faculty, Kingston University, Kingston-upon-Thames, United Kingdom; ^2^Health and Life Sciences, De Montfort University, Leicester, United Kingdom

**Keywords:** aldehydes, lipid oxidation products (LOPs), polydimethylsiloxane (PDMS), proton nuclear magnetic resonance (1H NMR), sunflower oil, thermo-oxidation

## Abstract

Suppressing the evolution of lipid oxidation products (LOPs) in commercially available culinary oils is considered to represent a valuable health-promoting incentive since these agents have cytotoxic and genotoxic properties and have been implicated in the pathogenesis of several chronic disease states. One agent used to suppress LOPs formation is polydimethylsiloxane (PDMS). In this study, proton nuclear magnetic resonance (^1^H NMR) analysis was employed to evaluating the influence of increasing PDMS concentrations (6.25 × 10^−7^, 1.0 × 10^−5^, 0.025, 0.05, 0.1, 0.5, 1.0, 5.0, and 10.0 ppm) in either stirred or unstirred refined sunflower oil exposed to thermal stressing episodes continuously at 180°C for 300 min with no oil replenishment. Results acquired showed that the extent of blockage of LOPs generation was correlated with increasing concentrations of PDMS. The minimal level of added PDMS required to provide a statistically significant protective role for both stirred and unstirred culinary oils when exposed to high frying temperatures was only 6.25 × 10^−7^ ppm. Furthermore, stirring at 250 rpm was experimentally determined to reduce the functional role PDMS. This is vital in a real world setting since the boiling process of frying may ultimately reduce the LOPs suppression activity of PDMS.

## Introduction

### Background

Lipid oxidation, in the presence of oxygen at standard frying temperatures (*ca*. 180°C) or higher, leads to the evolution of lipid oxidation products (LOPs), which are known to exert cytotoxic and genotoxic effects towards human cells (1, 2). Classically, such LOPs include primary peroxidation products such as conjugated hydroperoxydienes and epoxy-fatty acids, along with their secondary fragmentation products, which include a range of lower-molecular-mass agents, notably toxic aldehydes, both saturated and unsaturated. Culinary oils which are employed for high-temperature frying episodes include those which are polyunsaturated-, monounsaturated- or even saturated fatty acid-rich classes, and in the Western world typically PUFA-rich sunflower, corn, soybean oils will be used for commercial restaurant frying purposes, and/or oil blends containing mixtures of differing vegetable-derived culinary oils, including rapeseed or canola oils, which alternatively contain quite high MUFA contents.

The degree of unsaturation of frying oils critically determines their susceptibility to thermo-oxidation, and markedly increases with increasing numbers of –CH=CH– units available in their molecular structures, with polyunsaturated fatty acids (PUFAs) being more highly prone to thermo-oxidation than monounsaturated fatty acids (MUFAs), which in turn are much more susceptible to oxidation than saturated fatty acids (SFAs). However, since PUFA-rich cooking oils constitute a rich source of LOPs, their continued regular and continuous use for frying purposes, domestic or commercial, presents some serious public health risks as have been reported in fried food sources (2–6). Comparatively, peroxidation-resistant MUFAs and SFAs, which are the predominant classes of FAs present in olive and coconut oils, respectively, generate much lower levels of LOPs, and only limited numbers of structurally less complex and less toxic aldehyde classes, i.e., *trans*-2-alkenals [also known as (*E*)-2-alkenals] and *n*-alkanals only (1, 2, 7–9).

Notably, chemical agents, both naturally present and/or synthetically designed to suppress and/or prevent the evolution of LOPs in culinary oils are currently being exploited by scientists from geographically, diverse backgrounds across the globe (10–14). One of these agents, which is yet to be extensively studied, is polydimethylsiloxane (PDMS). PDMS is odourless, colourless and transparent, and its heat-stability is largely dependent on its nominal kinematic viscosity. Being a silicone-based polymer, low concentrations of PDMS are used extensively as an anti-foaming agent for frying oils, and since it and its derivatives also form an O_2_-impermeable layer on culinary oil surfaces, they have the ability to enhance the resistivities of such oils against thermo-oxidative damage (15). However, as with the frying oils themselves, PDMS is incorporated into food matrices available for human ingestion when they are fried or cooked in PDMS-treated culinary oils (16).

The nature and extent of PDMS's ability to protect against the peroxidation of unsaturated fatty acids (UFAs) in culinary frying oils has been previously evaluated using classical standard experimental techniques, e.g., high-performance size exclusion chromatography (15), oxygen monitoring with a biological oxygen monitor (17), linoleoylglycerol degradation by gas chromatography (GC), tocopherol quantification by high-performance liquid-chromatography (HPLC), and determinations of the secondary oxidation product 4-hydroxy-2-(*E*)-nonenal by GC-electron impact mass spectrometry (MS) (18). However, these techniques are generally targeted, and are focused on only one or several analyte compounds only; moreover, several of them have a limited specificity, and are laborious and time-consuming to perform.

High-resolution nuclear magnetic resonance (NMR) analysis is a highly efficient and reliable technique, which allows the identification and quantification of a very wide range of compounds in frying oils simultaneously. Furthermore, important recent applications such as Pure Shift Yielded by Cherp Excitation (PSYCHE), and hyphenated diffusion techniques (PSYCHEiDOSY), in high-field NMR analysis allows for the resolution of small molecule resonances when present in complex multicomponent mixtures (19). In this report, a PUFA-rich culinary oil (sunflower oil), which has been shown experimentally to be highly susceptible to thermo-oxidation (3), was exposed to a continuous thermo-oxidation episode at 180°C, both with and without increasing concentrations of added PDMS. The influence of magnetic stirring agitation on the susceptibility of this common frying oil to peroxidation, and on the ability of PDMS to suppress this undesirable process, was also explored. This research builds on the impact of continuous and discontinuous thermo-oxidation on the evolution of LOPs in culinary oils (3) and the “real-world” potential consequences of such cytotoxic and genotoxic LOPs in French fries fried in reheated culinary oils over prolong period sourced from two global chain fast-food restaurants (6).

### Rationale

This research investigation seeks to address the following key questions:

1. How do increasing concentrations of added PDMS protect UFAs in sunflower oil against thermo-oxidation continuously at 180°C for a 300 min duration?
2. To what degree does the stirring process influence the molecular nature and concentrations of LOPs in sunflower oil thermally stressed in this manner without an oil replacement strategy?

## Materials and Methods

### Culinary Oil Product Investigated

Sunflower oil samples were procured from a local supermarket in London, United Kingdom. The oil was stored in the dark in a cool, dry room at ambient temperature for not more than 72 h. until experimental analysis was conducted. The fatty acid composition was specified as 89.13% total UFAs, of which 60.87 and 28.26% were PUFAs and MUFAs, respectively, and 10.87% SFAs. This class of cooking oil was selected for these studies since it is one of most highly peroxidatively-susceptible frying oils available, and therefore generates high levels of toxic LOPs in response to thermo-oxidation induced by high frying temperatures (3). This arises from its very high content of PUFAs.

### Thermo-Oxidation of Sunflower Oil Samples

Thermal stressing of sunflower oil at 180°C was conducted over a 300 min period in the presence of atmospheric O_2_. Three replicate samples of 20.0 g quantities sunflower oil were accurately weighed on an electronic balance [±0.10 g accuracy, Mettler (UK), Model AT261] and were then thermally stressed in 100 mL beakers of the same size and type using an electronically controlled hot-plate operating at 230 V, 50 Hz, and 750 W (Model SB162, Stuart heat-stir, UK). The oil-air surface area of sunflower oil in the 100 mL beaker (of surface area 149.23 cm^2^) was 39.27 cm^2^. Thermo-oxidation of sunflower oil was performed in a continuous manner with no intermittent cooling and no oil replacement processes in place. Sampling of oil samples was performed at 60 min intervals for a total duration of 300 min.

### PDMS Treatment

According to Gerde et al. (17), the minimal level of PDMS required to form a satisfactory monolayer on cooking oils is equivalent to a final concentration of 25 ppb; concentrations lower than this are viewed to be ineffective. The concentration of PDMS in a typical frying medium is computable from equation 1 and the result is expressed in ppb (17).

(1) $$\begin{array}{l} \begin{array}{c} PDMS\;Concentration \end{array}\\ \;\begin{array}{c} =\frac{Area\;of\;Container\;\times \;MW\;of\;Monomer}{Area\;of\;Monomer\;\times \;NA\;\times \;Mass\;of\;oil} \end{array} \end{array}$$

Where the area of container is 7.85 × 10^17^ Å^2^, area of PDMS monomer is 20 Å^2^, molecular weight (mw) of PDMS monomer is 7.41 × 10^10^ ng/mol, Avogadro's number (N_A_) is 6.022 × 10^23^, and the mass of oil is 200 g (17).

This present study sought to test the above hypothesis by working with a broader range of PDMS concentrations than those previously investigated. Concentrations of 10.0, 5.0, 1.0, 0.5, 0.1, 0.05, 0.025, 1.0 × 10^−5^, and 6.25 × 10^−7^ ppm of PDMS were prepared from the 100 ppm stock solution, and these concentrations were transferred to ensure that they fully covered the surface of 20 g sunflower oil in a 100 mL volume beaker. The PDMS-treated sunflower oil was then thermally stressed after the *n*-hexane solvent had evaporated.

All the above concentrations of PDMS were first tested in unstirred sunflower oil. Following that, a 300 min continuous thermo-oxidation process with automatic magnetic stirring was also investigated. Using a 12 × 3mm magnetic stirrer bar (Fisher Brand), PDMS-treated sunflower oil was stirred at 250 rpm on the bottom-side of the beaker. This generated a bubbling effect or frothing in the thermally stressed oil, and this technique was critical to mimic the bubbling effect observed when frying food in culinary oil. Stirring the oil-PDMS media was mainly focused on added PDMS concentrations of 10.0, 5.0, 1.0, and 0.5 ppm, and all experiments were conducted simultaneously. Sampling and preparation of thermally stressed culinary oil samples for ^1^H NMR analysis is described below in section Sampling and preparation of thermally stressed sunflower Oil.

### Sampling and Preparation of Thermally Stressed Sunflower Oil

Sampled oils were subjected to ^1^H NMR analysis using a Bruker Ultrashield 600 spectrometer (Kingston University London, London, UK) operating at 600.13 MHz frequency and 298 K probe temperature. The acquisition parameters were: 65,536 fids; 256 scans; probe temperature 300 K; spectral width 20.573 ppm; relaxation delay 1.000 s; acquisition time 4.819 s; pulse width 90°C; and total acquisition time 16 min (3).

A 0.30 mL aliquot of each sampled oil was diluted with 0.60 mL of deuterated chloroform (C^2^HCl_3_) (99.8% purity). A 0.50 mL aliquot of the resulting mixture was then thoroughly mixed with a 0.10 mL volume of the 1,3,5-tribromobenzene (TBB) quantitative internal standard (prepared by dissolving 4.14 mg TBB in 2.0 mL C^2^HCl_3_) in a 5-mm diameter NMR tube (Norrell HT, GPE Scientific). All signal resonances were referenced to tetramethylsilane (TMS) (δ = 0.000 ppm), residual C^2^HCl_3_ (δ = 7.283 ppm) and TBB (δ = 7.537 ppm). In addition, TBB served as an internal quantitative ^1^H NMR standard that was employed for determining ^1^H NMR-detectable LOPs in sampled sunflower oils (3).

#### Analysis of Acyl Groups and Iodine Value

The molar percentages of key fatty acyl (FA) groups and iodine values determined from the ^1^H NMR spectra of the oil samples were according to Guillén and Uriarte (20); Le Gresley et al. (3).

#### Analysis of Lipid Oxidation Products

All ^1^H NMR detectable LOPs analysed in the studied sunflower oil samples were identified with the aid of literature references (3, 4, 6, 21–24). The respective chemical shifts and multiplicities of the signal resonances of all identified LOPs in the studied oil samples were consistent with those published in the references cited above. Among all LOPs identified in the oils, only aldehydic LOPs were quantified. This was according to the formula proposed in the Supplementary Material of the publication by Le Gresley et al. (3).

### Experimental Design and Statistical Analysis

The experimental design for this study was classified as a 3-factor analysis-of-variance (ANOVA) system with added PDMS levels (P_*i*_), sampling time-points (T_*j*_) and stirring status (S_*k*_) representing fixed qualitative explanatory effects at 9, 6, and 2 levels, respectively [the between-time-points effect was included as a qualitative rather than a quantitative variable in view of the non-linearity of relationships between all aldehyde concentrations and heating times at 180°C; this is a major requirement assumption for analysis-of-covariance (ANCOVA)]. Also incorporated in the model were the three first-order PDMS level × time-point (PT_*ij*_), PDMS level × stirring status (PS_*ik*_) and time-point × stirring status (TS_*jk*_) interaction effects. The mathematical model for this experimental design is shown in equation 2, where y_ijkl_, μ and e_ijkl_ represent output variable observations (individual aldehyde concentrations), the mean value in the absence of all contributory sources of variation, and fundamental error, respectively. This univariate ANOVA analysis was performed for each of the 11 aldehyde analytes individually.

(2) $$\begin{array}{c} {\text{y}}_{ijkl}=\mu +{\text{P}}_{i}+{\text{T}}_{j}+{\text{S}}_{k}+{\text{PT}}_{ij}+{\text{PS}}_{ik}+{\text{TS}}_{jk}+{\text{e}}_{ijkl} \end{array}$$

## Results

### Characterisation of ^1^H NMR Spectra of Unheated Culinary Oils

#### Major Acyl Groups

**Figure 1:**
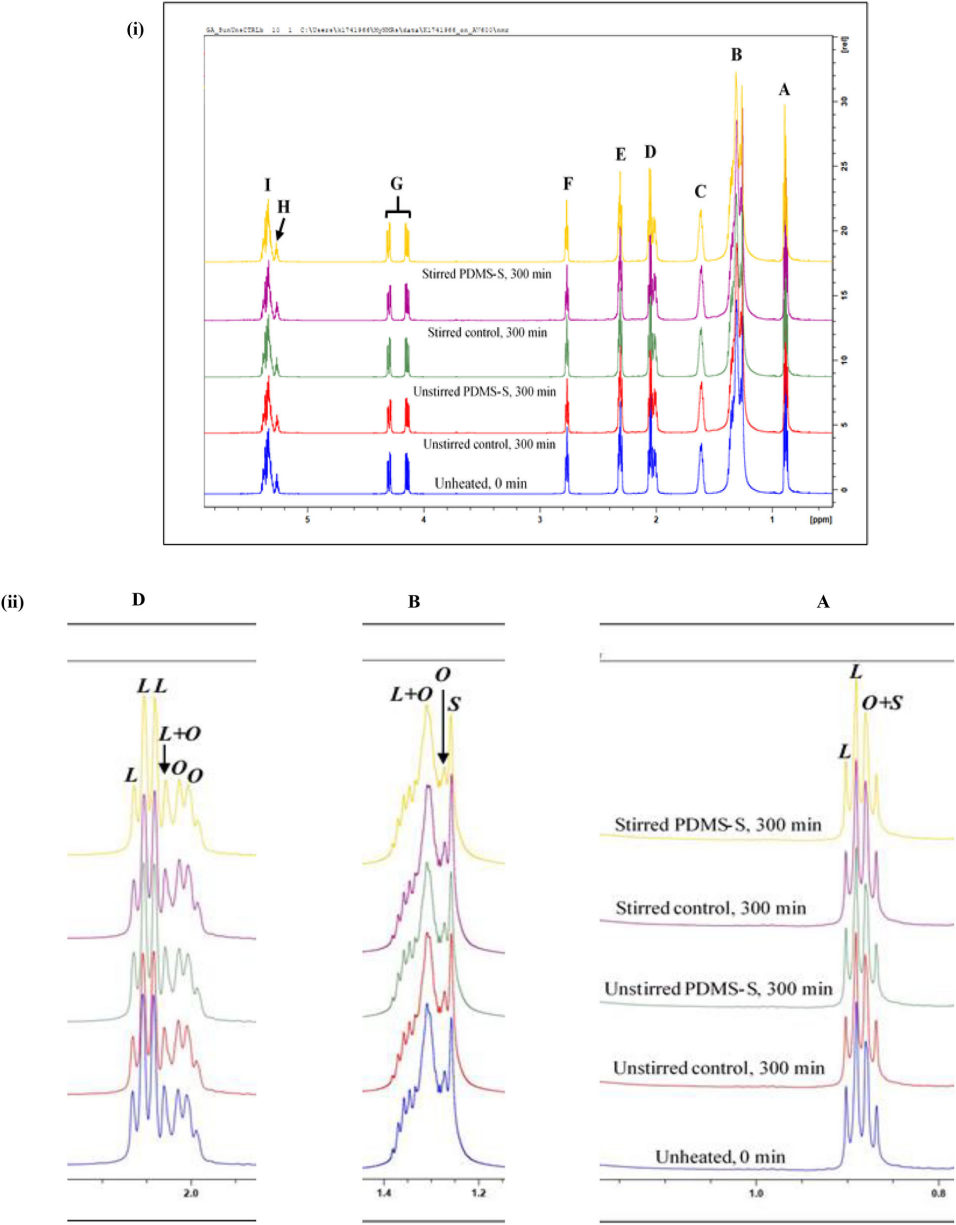
^1^H NMR spectra showing **(i)** major acylglycerol functions, and **(ii)** expanded regions of resonances A, B and D present within the 0.0–5.8 ppm regions of PDMS-treated sunflower oil thermally stressed continuously throughout a 300 min duration, expanded regions of resonances A, B, and D present within the 0.0–2.3 ppm spectral range of PDMS-treated sunflower oil thermally-stressed continuously throughout a 300 min duration. PDMS-S, Polydimethylsiloxane-treated sunflower oil; *L*, Linoleoylglycerol part-signal; *O*, oleoylglycerol part-signal; *S*, saturated fatty acid acylglycerol part-signal. Letter assignments of resonances correspond to those provided in Table 1.

Several sunflower oil types may vary somewhat in the degree of unsaturation; notwithstanding, the characteristic thermo-resistibilities of sunflower oils of any source will largely depend on their (PUFA):(MUFA) content ratios, along with the conditions of the frying process, e.g., temperature, duration, oil reuse extent, shallow- vs. deep-frying processes, foods fried and their water contents, etc. (3, 20). A typical ^1^H NMR spectrum of neat (unheated) sunflower oil evaluated in the current study, and that of a sample continuously thermally-stressed at a temperature of 180°C for a 300 min period is shown in Figure 1 full assignments of the resonances therein are provided in Table 1. Characteristic resonance assignments of the acyl chain functions are in accordance with those reported by Guillén and Uriarte (20); Percival et al. (25).

**Table 1:** Assignment of bulk ^1^H NMR signals of major acylglycerol groups present in the ^1^H NMR profiles of PDMS-treated sunflower oil, including chemical shift values, multiplicities, and their associated functional groups.

			**Functional group**	
**Signal**	**δ-scale (ppm)**	**Multiplicity**	**Condensed function**	**Classification**
A	0.836–0.929	*t*	–C**H**_3_	Saturated, oleoyl and linoleoyl acyl groups
B	1.201–1.407	*t*	–(C**H**_2_)*_*n*_*–	All acyl groups
C	1.569–1.662	*m*	–OCO–CH_2_-C**H**_2_-	Acyl groups except for those of DHA, EPA and ARA
D	1.975–2.089	*m*	–C**H**_2_-CH-CH–	All acyl groups except for DHA
E	2.271–2.357	*dt*	–OCO–C**H**_2_-	All acyl groups except for DHA
F	2.743–2.798	*t*	-HC–C**H**_2_-CH-	Diunsaturated ω-6 acyl groups
G	4.109–4.336	*dd, dd*	–C**H**_2_OCOR	Glyceryl backbone groups
H	5.237–5.292	*m*	>C**H**OCOR	Glyceryl backbone groups
I	5.292–5.416	*m*	–C**H**-C**H**–	Acyl chain olefinic functions

*d, doublet; t, triplet; m, multiplet; dd, doublet of doublets; dt, doublet of triplets; ω-6, omega-6 acyl groups; DHA, docosahexaenoyl acyl groups; EPA, eicosapentaenoyl acyl groups; ARA, arachidonoyl acyl groups. Letter assignments and characteristics correspond to those provided in Figure 1*.

Accordingly, signal A is a triplet located at 0.836–0.929 ppm and is ascribable to the terminal methylic functions (–C**H**_3_) of all saturated, oleoyl and linoleoyl acyl groups [Figure 1(i)]. When expanded, the linoleoylglycerol component (*L*) of signal A showed a higher intensity than that of the composite oleoylglycerol/saturated fatty acid acylglycerol signal (*O* + *S*) [Figure 1(ii)A]. Signal B (δ = 1.201–1.407 ppm in all sunflower oil spectra) is a triplet ascribable to the bulk methylene protons (–(C**H**_2_)*n*–) of all acyl groups [Figure 1(i)]. Expanding signal B revealed that the composite linoleoylglycerol and oleoylglycerol component (*L* + *O*) to be of the highest intensity when compared to that of the saturated fatty acid acylglycerol (*S*) and oleoylglycerol (*O*) ones, in that order [Figure 1(ii)B]. The higher intensity of *L* observed for both signals A and B is, of course, a reflection of the high unsaturation degree of sunflower oil.

Located at 1.569–1.662 ppm is signal C, which is a multiplet assigned to β-substituted methylenic protons (–OCO–CH_2_-C**H**_2_-) of all acyl groups, except for those of docosahexaenoyl (DHA), eicosapentaenoyl (EPA) and arachidonoyl (ARA) groups, all of which have little or no contents in sunflower oil [Figure 1(i)]. Signal D is also a multiplet, however, signal D is assigned to the *mono*-allylic protons (–C**H**_2_-C**H**-C**H**–) of all acyl groups [Figure 1(i)]. When expanded, signal D of sunflower oil comprised contributions from *L, O* and *L* + *O* components [Figure 1(ii)D]. Overall, in agreement with the expanded chemical shift range (δ-scale) of signals A and B, the *L* fraction of signal D was characterised by higher intensities that were expressed over *O* and *L*+*O* [Figure 1(ii)D]. The expanded δ-scale of signals A, B, and D are consistent with the findings of Guillén and Uriarte (20).

Signal E of the ^1^H NMR spectra of sunflower oil is a doublet of triplets located at 2.271–2.357 ppm which is ascribable to the methylenic protons (–OCO–C**H**_2_-) of all acyl groups except for that of DHA [Figure 1(i)]. The *bis*-allylic protons (-HC–C**H**_2_-CH-) of linoleoyl acyl group resonance is labelled as signal F, which can be observed as an apparent triplet at δ = 2.743–2.798 ppm of the ^1^H NMR spectrum of sunflower oil [Figure 1(i)]. Signal G is a pair of doublet of doublets signals arising from the protons located on carbon atoms 1 and 3 of the glyceryl backbone (–C**H**_2_OCOR). Complementarily, the multiplet multiplicity of signal H, at 5.237–5.292 ppm, is derived from protons on carbon atom 2 of the glyceryl group (>C**H**OCOR) [Figure 1(i)]. Signal I, which is key in determining the overall unsaturation degree of culinary oils in general, is ascribable to olefinic protons (–C**H**-C**H**–) of several acyl groups [Figure 1(i)]. The key acyl group resonances were integrated and quantified, and modifications to these were reported in section Results.

#### Minor Compounds Detectable

**Figure 2:**
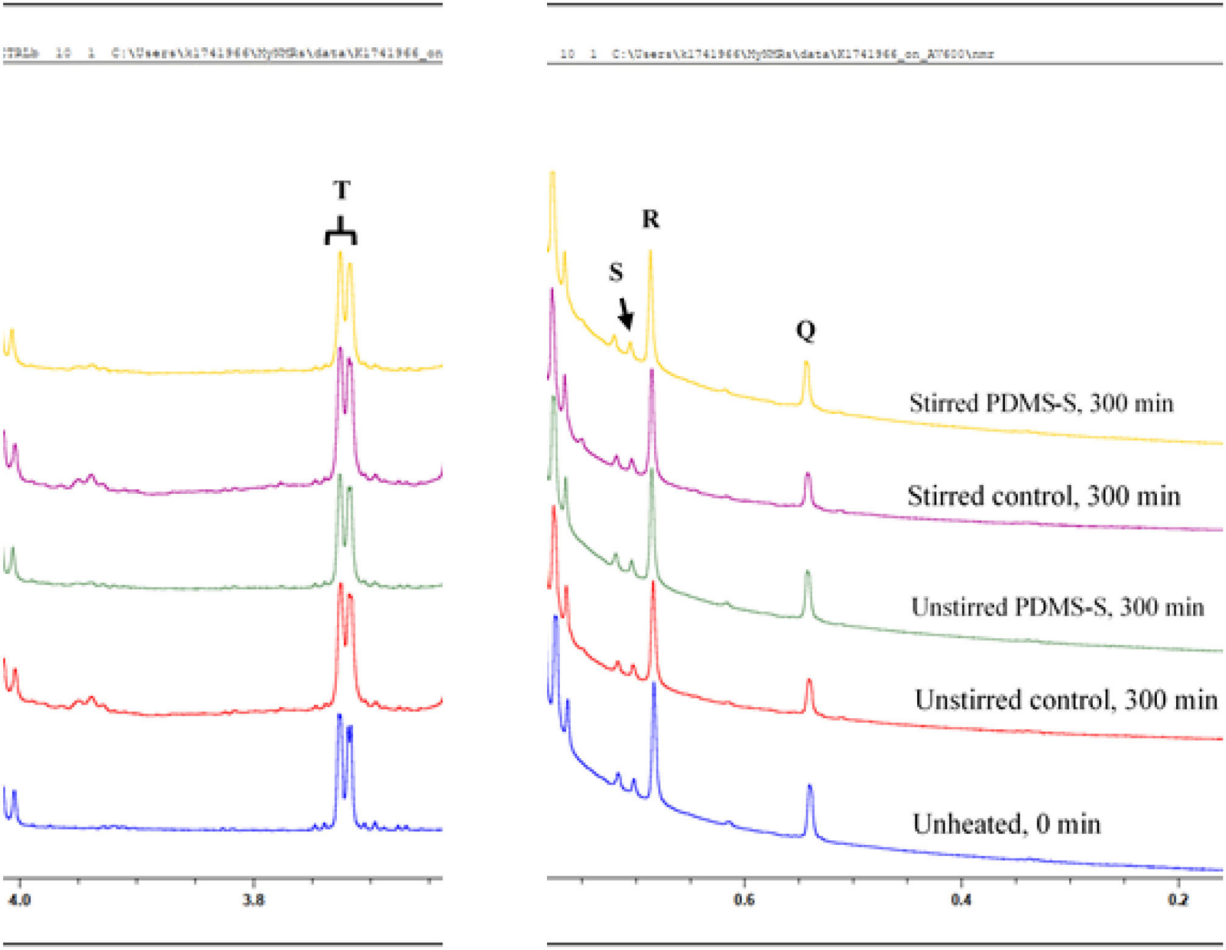
^1^H NMR spectra of minor compounds detectable in the 0.4–4.0 ppm regions of PDMS-treated sunflower oil thermally stressed continuously throughout a 300 min duration. PDMS-S, Polydimethylsiloxane-treated sunflower oil. Letter assignments of resonances correspond to those provided in Table 2.

Minor compounds, which may be naturally present in culinary oils, may. at least in principle, play significant roles in influencing the susceptibility of frying oils to thermo-oxidation, in addition to offering possible nutritional benefits to consumers. All minor compounds identified in the sunflower oil product investigated here by ^1^H NMR analysis are shown in Figure 2, which reveals the expanded regions of the signal resonances within 0.4–0.8 ppm and 3.7–3.8 ppm regions of the ^1^H NMR profiles acquired. In view of their low concentrations, which are reflected by their low resonance intensities, these minor compounds are classified under Generally Recognised As Safe (GRAS) agents (26–28). The general characteristic of each ^1^H NMR detectable minor compound is further highlighted in Table 2. The assignment of the ^1^H NMR signals of the minor compounds identified in this study are consistent with those reported by Martínez-Yusta et al. (23); Percival et al. (25).

**Table 2:** Assignment of bulk ^1^H NMR signals of minor compounds present in the ^1^H NMR profiles of PDMS-treated sunflower oil, including chemical shift values, multiplicities, and their associated functional group assignments.

			**Functional group**	
**Signal**	**δ-scale (ppm)**	**Multiplicity**	**Condensed function**	**Classification**
Q	0.533–0.546	*s*	–C**H**_3_ (C-18)	Δ7-Avenasterol
R	0.677–0.690	*s*	–C**H**_3_ (C-18)	β-Sitosterol, Δ5-Campesterol (or C-18 –C**H**_3_ Cholesterol)
S	0.697–0.706	*s*	–C**H**_3_ (C-18)	Δ5-Stigmasterol and Brassicasterol
T	3.705–3.730	*d*	–C**H**_2_OH	1,2-Diacylglycerols

*s, singlet; d, doublet. Letter assignments and characteristics correspond to those provided in Figure 2*.

Signal Q, a singlet at 0.533–0.546 ppm is ascribable to the C-18 –C**H**_3_ function of Δ7-avenasterol (Figure 2). Signal R, also a singlet and positioned at 0.677–0.690 ppm may be ascribable to the C-18 –C**H**_3_ of β-sitosterol, Δ5-campesterol or cholesterol (Figure 2). Signal S is a singlet positioned at 0.697–0.706 ppm of the ^1^H NMR spectra of sunflower oil. Signal S is assigned to the C-18 –C**H**_3_ of Δ5-stigmasterol and brassicasterol (Figure 2). Unlike signals Q, R and S, signal T is a doublet located at 3.705–3.730 ppm (Figure 2). Signal T is ascribable to the protons directly attached to the primary carbon (–C**H**_2_OH) of 1,2-diacylglycerols, which are chemically produced from triacylglycerols through the direct hydrolysis of triacylglycerols.

It should also be noted that the signal resonances of PDMS, which is supposed to be a singlet at 0.042 ppm, was not observable on any of the ^1^H NMR spectrum examined for sampled PDMS-treated sunflower oil. This may be ascribable to the lack of miscibility or limited solubility of PDMS with the studied sunflower oil. Furthermore, the size of the PDMS employed in the present study may results in its signal peak broadening, which could make it difficult to identify and characterise its signal peak on the spectra of the PDMS-treated sunflower oil.

### Evolution of LOPs

#### Primary LOPs

**Figure 3:**
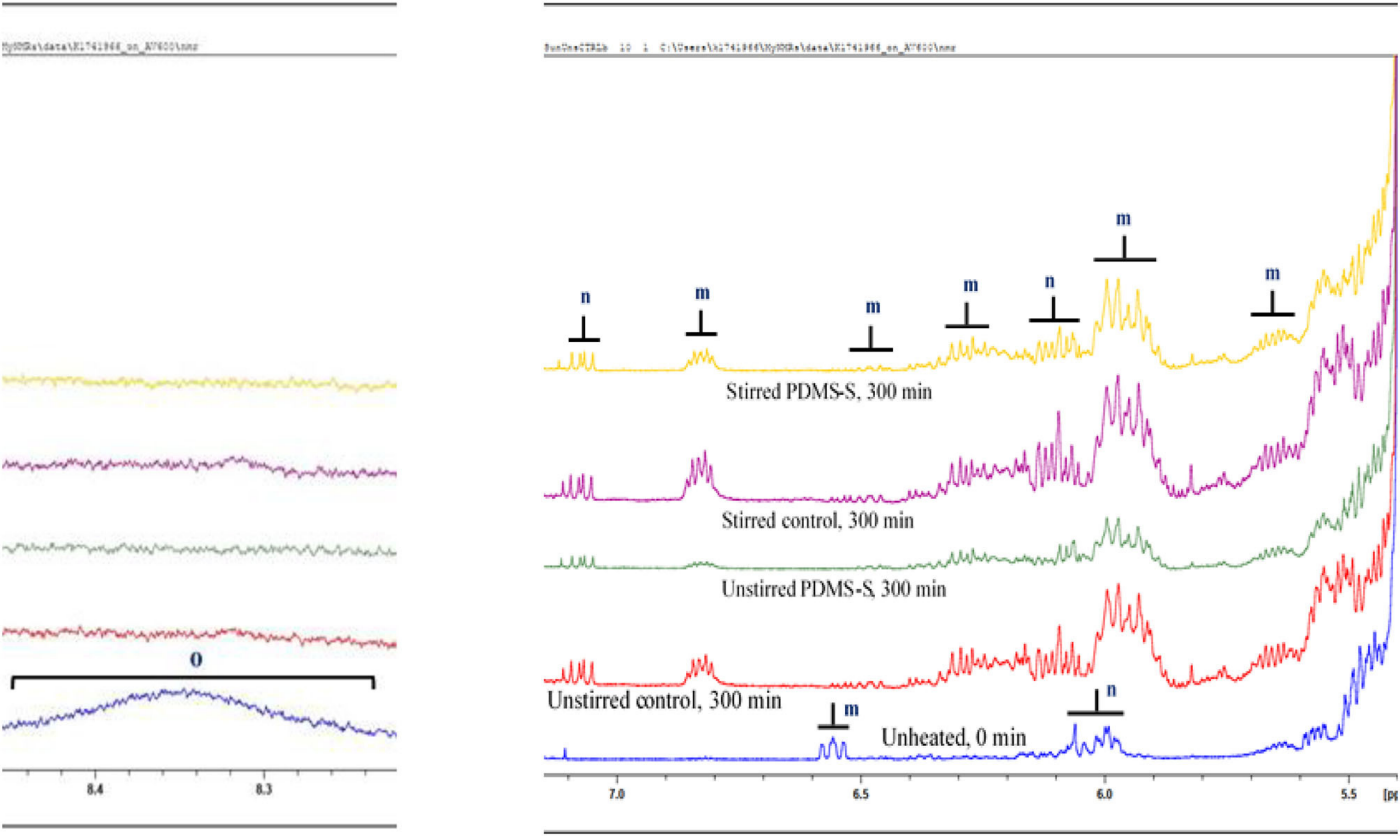
^1^H NMR spectra showing expanded 5.5–7.0 ppm regions of PDMS-treated sunflower oil thermally stressed continuously throughout a 300 min duration. The expanded ^1^H NMR profiles reveal conjugated diene hydroperoxydienes, and hydroxymonoenes (both primary LOPs), and the olefinic resonances of α,β-unsaturated aldehydes. Broad resonances attributable to -OOH group protons present in the 8.2–8.6 ppm regions of PDMS-untreated, unheated (0 min) sunflower oil are also shown. PDMS-S, Polydimethylsiloxane-treated sunflower oil. Letter assignments of resonances correspond to those provided in Table 3.

Conjugated hydroperoxydienes (CHPDs) and hydroxymonoenes and olefinic resonances of alpha, beta-unsaturated aldehydes (α,β-UAs) were the primary LOPs identified in sunflower oil (Table 3; Figure 3). Two kinds of (*E,E*)-conjugated olefinic protons of CHPDs were detected in unheated sunflower oil. All identified signals of primary LOPs became prominent in a prolong continuous thermo-oxidation of both stirred and unstirred sunflower oil, including PDMS-treated sunflower oil (Figure 3). This was evidenced in the intensity, which is also a representation of the concentrations of the signals of primary LOPs. Comparatively, PDMS-treated sunflower oils showed lower signal intensities of primary LOPs than their respective control experiments. Unstirred PDMS-treated sunflower oil however, demonstrated a greater suppression of primary LOPs than stirred PDMS-treated sunflower oil. This was also true for the comparison between unstirred and stirred thermally stressed sunflower oil. Agitation or stirring increases the kinetic energy of reaction mixture and therefore, promotes oxygen solubility and chemical interactions of molecular oxygen with UFA. As a consequence, this leads to the evolution of LOPs. Primary LOPs are important intermediates since their evolution precedes secondary LOPs. Despite primary LOPs being undetectable in many studies, the present study employability of 600 MHz NMR, under the same processing conditions as that reported by Le Gresley et al. (3) demonstrated that they are trackable. This was also consistent with the findings of Guillén and Uriarte (20), Martínez-Yusta et al. (23) and Percival et al. (24).

**Table 3:** Assignment of ^1^H NMR signals of conjugated diene hydroperoxydienes and hydroxymonoenes, and olefinic resonances of α,β-unsaturated aldehydes present in the ^1^H NMR profiles of PDMS-treated sunflower oil, including chemical shift values, multiplicities, and their associated functional group assignments.

			**Functional group**	
**Signal**	**δ-scale (ppm)**	**Multiplicity**	**Condensed function**	**Classification**
*m*	5.595–5.704	*ddm*	–C**H**-C**H**–C**H**-C**H**–	(*E,E*)-conjugated olefinic protons of CHPDs
*m*	5.863–6.024	*ddm*	–C**H**-C**H**–C**H**-C**H**–	
*m*	6.236–6.345	*ddd*	–C**H**-C**H**–C**H**-C**H**–	
*m*	6.431–6.565	*ddtd*	–C**H**-C**H**–C**H**-C**H**–	
*m* ^*^	6.525–6.589	*ddd*	–C**H**-C**H**–C**H**-C**H**–	
*m*	6.782–6.869	*ddm*	–C**H**-C**H**–C**H**-C**H**–	
*n* ^*^	5.957–6.088	*dddm*	–C**H**-C**H**–C**H**-C**H**–	(*Z,E*)-conjugated olefinic protons of CHPDs
*n*	6.042–6.141	*ddm*	–C**H**-C**H**–C**H**-C**H**–	
*n*	7.037–7.099	*dddd*	–C**H**-C**H**–C**H**-C**H**–	
*o* ^*^	8.200–8.600	-	-OO**H**	Hydroperoxide groups

* *Distinctly identified only in the control experiment, which is PDMS-untreated, unheated (0 min) sunflower oil. CHPDs, conjugated hydroperoxydienes; d, doublet; t, triplet; m, multiplet; dd, double doublet. Letter assignments of resonances correspond to those provided in Figure 3. Major, quite clearly visible CHPD signals are at ca. 6.0 and 6.5 ppm for (Z,E)-CHPDs, and ca. 6.25 and 6.75 ppm for (E,E)-CHPDs*.

#### Secondary LOPs

**Figure 4:**
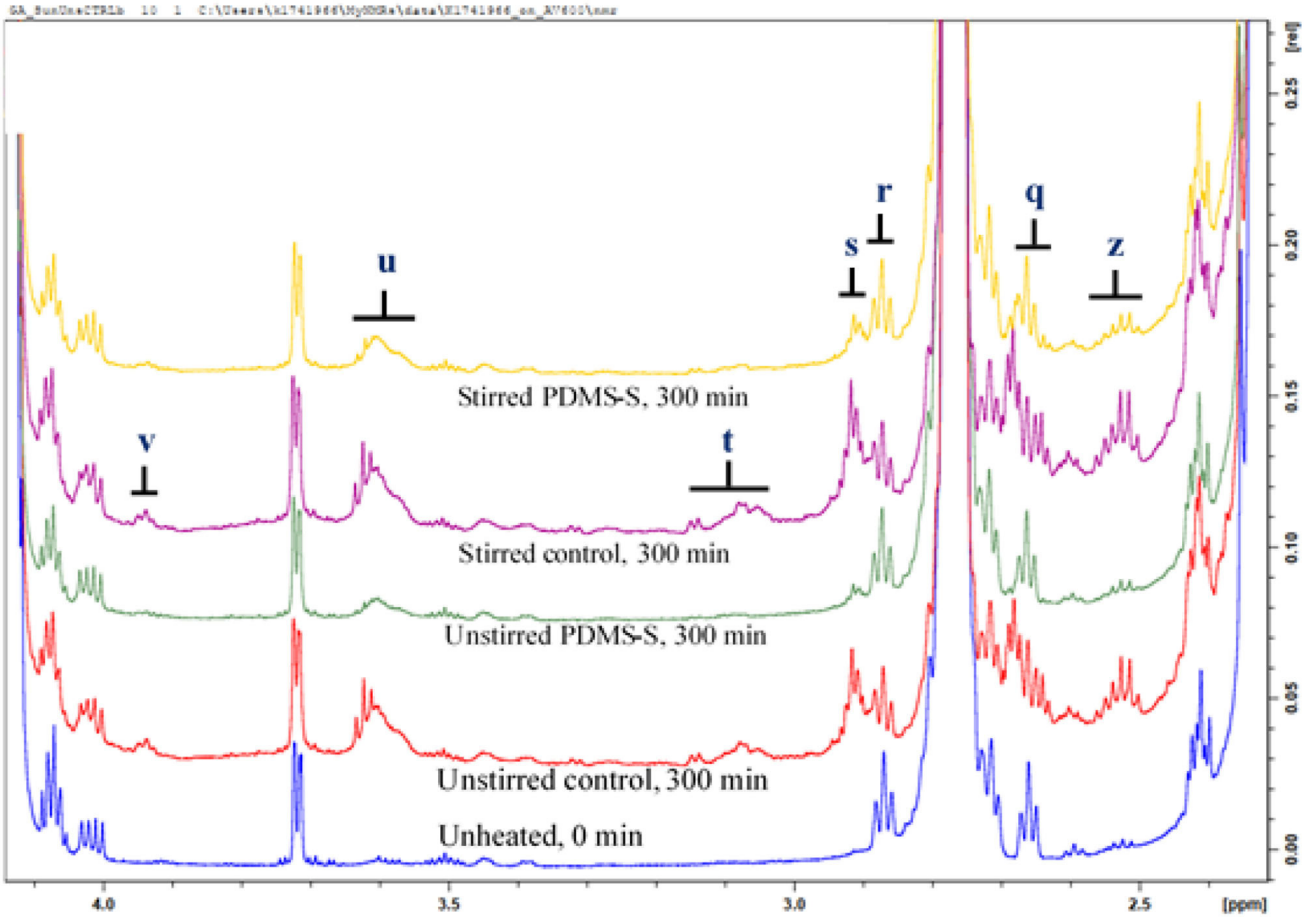
^1^H NMR spectra showing epoxides and primary alcohols present in the expanded 2.4–4.0 ppm regions of PDMS-treated sunflower oil thermally stressed continuously throughout a 300 min duration. PDMS-S, Polydimethylsiloxane-treated sunflower oil. Letter assignments of resonances correspond to those provided in Table 4.

**Figure 5:**
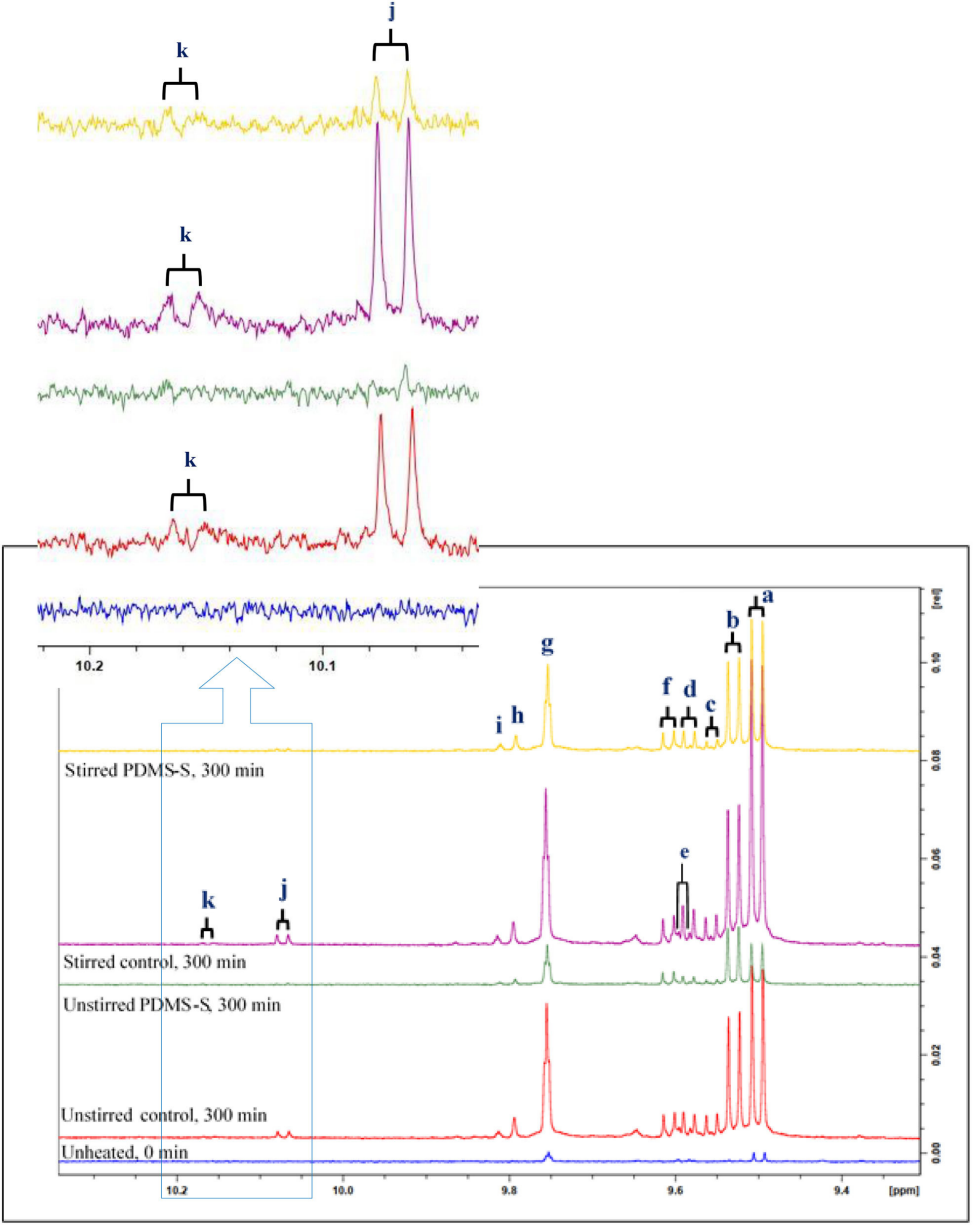
^1^H NMR spectra showing aldehydes detectable in the expanded 9.4–10.2 ppm regions of an untreated and PDMS-treated sunflower oil product exposed to a thermal stressing episode continuously throughout a 300 min duration. PDMS-S, Polydimethylsiloxane-treated sunflower oil. Letter assignments of resonances correspond to those provided in Table 5.

Secondary LOPs constitute the central focus of this report in view of their adverse toxicological properties. The secondary LOPs identified in this reported are presented in Table 4 and Figure 4 (epoxides and primary alcohols) and Table 5 and Figure 5 (aldehydes). It is also worth noting that epoxides can be considered as either primary or secondary, dependent on their route of formation.

**Table 4:** Assignment of ^1^H NMR signals of epoxides and primary alcohols present in the ^1^H NMR profiles of PDMS-treated sunflower oil, featuring chemical shift values, multiplicities, and their associated functional group assignments.

			**Functional group**	
**Signal**	**δ-scale (ppm)**	**Multiplicity**	**Condensed function**	**Classification**
*z*	2.491–2.565	*m*	-	Unidentified
*q*	2.622–2.696	*m*	–C**H**O**H**C–	(*E*)-9,10-Epoxystearate
*r*	2.849–2.892	*m*	–C**H**O**H**C–	(*Z*)-9,10-Epoxystearate
*s*	2.892–2.932	*m*	–C**H**OHC–CHO**H**C–	9,10–12,13-Diepoxyoctadecanoate
*t*	3.026–3.157	*m*	–C**H**O**H**C–	9,10-Epoxy-octadecanoate; 9,10-Epoxy-12-octadecenoate (leukotoxin); and 12,13-Epoxy-9-octadecenoate (isoleukotoxin)
*u*	3.543–3.641	*m*	–CHO**H**C–CH_2_-C**H**OHC–	9,10–12,13-Diepoxyoctadecanoate
*v*	3.918–3.955	*m*	*α*-C**H**_2_	Primary alcohol LOPs

*m, multiplet. Letter assignments of resonances correspond to those provided in Figure 4*.

**Table 5:** Assignment of –CHO functional groups of ^1^H NMR signals of aldehydic LOPs (secondary LOPs) present in the ^1^H NMR profiles of PDMS-treated sunflower oil, including chemical shift values, multiplicities, and their associated functional group assignments.

			**Functional group**	
**Signal**	**δ-scale (ppm)**	**Multiplicity**	**Condensed function**	**Classification**
*a*	9.483–9.513	*d*	–C**H**O	(*E*)-2-Alkenals
*b*	9.513–9.541	*d*	–C**H**O	(*E,E*)-2,4-Alkadienals
*c*	9.541–9.563	*d*	–C**H**O	4,5-Epoxy-(*E*)-alkenals
*d*	9.569–9.590	*d*	–C**H**O	4-Hydroxy-(*E*)-2-alkenals
*e*	9.577–9.594	*d*	–C**H**O	4-Hydroperoxy-(*E*)-2-alkenals
*f*	9.594–9.616	*d*	–C**H**O	(*Z,E*)-2,4-Alkadienals
*g*	9.741–9.762	*t*	–C**H**O	*n*-Alkanals
*h*	9.784–9.800	*t*	–C**H**O	4-Oxo-alkanals
*i*	9.800–9.816	*t*	–C**H**O	*n*-Alkanals of low-molecular-mass (ethanal, propanal and butanal)
*j*	10.056–10.080	*d*	–C**H**O	(*Z*)*-*2-Alkenals
*k*	10.144–10.170	*d*	–C**H**O	Unidentified unsaturated aldehyde

*d, doublet; t, triplet. Letter assignments of resonances correspond to those provided in Figure 5*.

##### Primary Alcohols

According to Figure 4, signal *v*, a multiplet is located at 3.918–3.955 ppm in the ^1^H NMR spectra of sunflower oil, is attributed to the α-C**H**_2_ protons of primary alcohols. The intensity of signal *v* increased with the thermo-oxidation duration of sunflower oil, as expected. At a constant temperature of 180°C, and under the same experimental conditions, the intensity of signal *v* was higher in stirred sunflower oil than in unstirred sunflower oil. PDMS-treatment of sunflower oil, however, considerably suppressed the intensity of signal *v* when compared to that of the control samples (Figure 4). Unheated sunflower oil did not, however, contain any traces of primary alcohols. This observation is consistent with the findings of Le Gresley et al. (3) and Martínez-Yusta et al. (23).

##### Epoxides

According to the review by Martínez-Yusta et al. (23), epoxides evolve from the thermo-oxidation of oleoyl- and linoleoylglycerol acyl chains. Typically, those two chains constitute the highest levels of UFAs present in sunflower oil, with greater amounts of the latter. The evolution of epoxides at constant temperature (180°C) accorded with the thermo-oxidation duration of sunflower oil. Similar to the trend observed for primary LOPs, the decreasing order of magnitude of epoxide concentrations amongst the conditioned sunflower oils were stirred control > unstirred control > stirred PDMS-S > unstirred PDMS-S > unheated (0 min) (Figure 4). The letter assignments of resonances illustrated in Figure 4 correspond to those provided and assigned in Table 4. Depending on the oil unsaturation degree, frying conditions and foods fried, etc., this study provided a projection of what may occur during standard frying/cooking processes conducted at this temperature. The use of 10 ppm (maximum legal limit) of PDMS for frying episodes performed at 180°C suppressed the evolution of epoxides in view of its ability to “screen” the oil surface from exposure to atmospheric O_2_. Therefore, it appears that PDMS has the ability to substantially diminish the levels of thermally induced epoxides in culinary oils exposed to high temperature frying episodes.

##### Aldehydes

Aldehydes identified in this report are of a series of categories, and these include both saturated and α,β-unsaturated aldehydes (α,β-UAs). The signals arising from α,β-UAs and saturated aldehydes in the ^1^H NMR spectra acquired are doublets and triplets, respectively (Figure 5; Table 5). Between the two groups, α,β-UAs are more chemically-reactive and hence more toxic, and this is ascribable to the susceptibility of the unsaturated 3-position carbon in these agents to nucleophilic attack by thiolate and amino functions present in biomolecules.

Although unidentified, the *J*-value of signal *k* (8.20 Hz) suggest that it is likely to be α,β-UAs (Figure 5). Also, signal *j*, which was first identified by Moumtaz et al. (4), has been proven to arise from (*Z*)-2-alkenals (4). Notably, there was a significant superimpositions of resonances d and e (Figure 5). At a constant temperature of 180°C, the types and intensity of aldehydic LOPs increased with increasing duration of thermo-oxidation of this oil. For all LOPs investigated, stirred sunflower oil samples gave rise to the highest content of LOPs, and this was followed by corresponding unstirred samples. However, when compared to results acquired from these experiments, PDMS treatment was found to greatly suppress aldehydic oxidation product generation throughout the 300 min continuous thermo-oxidation period. Between PDMS-treated sunflower oils, stirred oil samples produced higher aldehydic oxidation product levels than those generated by unstirred PDMS-treated sunflower oils. The stirring technique employed in agitating sunflower oil in this report may be comparable to domestic or commercial frying or cooking methods. The LOPs suppression activity of PDMS prevented unidentified signal *k* from forming in 300 min thermally stressed sunflower oil which was otherwise produced in their respective control experiments. Notwithstanding, three α,β-UAs; (*E*)-2-alkenals (signal *a*), (*E,E*)-2,4-alkadienals (signal *b*) and (*Z,E*)-2,4-alkadienals (signal *f*), as well as one saturated aldehyde class (*n*-alkanals, signal *g*) were identified and quantified in purchased unheated sunflower oil.

## Discussion

### Thermo-Oxidation of Sunflower Oil

#### Chemical Modifications to Acyl Groups

^1^H NMR analysis revealed that the unheated sunflower oil product contained 30.04 ± 0.08% oleoyl acyl groups, 56.16 ± 0.15% linoleoyl acyl (PUFA) groups, 86.20 ± 0.09% total UFAs, and 13.80 ± 0.09% SFAs (mean ± SD values). When compared to the measurements stipulated on the product label, our ^1^H NMR data indicated that the oleic acyl/MUFA and SFA contents were ca. 1.8 and 2.9% higher, respectively, whereas linoleic acyl and PUFA groups were ca. 4.7 and 2.9% lower than the percentages stipulated on the product label. It is worth mentioning that the ^1^H NMR derivations provide molar percentage values, whereas those on the product label are w/w percentage. This explains the differences in data in the acyl groups stipulated on the product label and those derived from our ^1^H NMR measurements.

Thermo-oxidation of sunflower oil at 180°C for a 300 min, period increased oleoyl acyl groups and SFAs, as expected; simultaneously, decreased linoleoyl acyl groups, i.e., total PUFA and total UFA groups. This is because PUFAs are more susceptible to peroxidation than MUFAs, and MUFAs more so than SFAs. Therefore, PUFA levels go down, and MUFA and SFA levels go up simultaneously as a consequence of thermo-oxidation of sunflower oil. This was in agreement to the findings of the thermo-dynamic changes in the acyl groups of sunflower oil thermally stressed at varying durations at 190°C (20), 200°C (29), and 180°C (3).

Stirring sunflower oil at 180°C resulted in a considerably higher degradation of UFA groups, whilst it simultaneously increased the formation of S(+M)FA groups, when compared to unstirred thermally stressed sunflower oil (Supplementary Figure 1). By comparison, the thermo-oxidation of linoleic fatty acyl groups, PUFAs, and UFAs, as well as IV, were less profound in unstirred sunflower oil than in stirred sunflower oil—both without PDMS treatment (Supplementary Figure 1). It was also observed that the thermo-oxidation of UFAs of sunflower oil was inversely proportional to the formation of S(+M)FA groups (Supplementary Figure 1). However, changes in oleic fatty acyl groups were in no order although their levels increased in sunflower oil with a prolong thermo-oxidation duration (Supplementary Figure 1).

Under the same experimental conditions. these modifications were more profound in stirred sunflower oil at all added PDMS concentrations. Stirring increases the motion of unsaturated triacylglycerols and ensures a greater or more rapid dissolution of oxygen molecules in thermally stressed oils, a process facilitating enhanced reactivities in thermo-oxidative reactions. Traditionally, frying or cooking food may lead to a far greater change in UFAs, since moisture from food generates energetic bubbling that underscores higher oxygen solubility. Nonetheless, factors such as the fryer and frying (deep or shallow) type, frying duration and temperature, culinary oil unsaturation degree, as well as food moisture content, composition and surface area may influence such reactivities.

Polydimethylsiloxane (PDMS) offers concentration-dependent protective effects against thermo-oxidative changes in culinary oils when exposed to high temperature frying episodes. Amongst the nine studied PDMS concentrations tested in unstirred sunflower oil here, 0.05, 0.025, 1.0 × 10^−5^, and 6.25 × 10^−7^ ppm PDMS offered little or no protective effects (Supplementary Figure 1). However, although PDMS at an added level of 1.0 ppm was ineffective in suppressing the thermo-oxidation of MUFAs, it was successful in blocking the thermo-oxidation of linoleoylglycerols. This was also reflective in the overall unsaturation degree of unstirred sunflower oil, which is also defined by the oil's IV (Supplementary Figure 1). Unlike the above PDMS concentrations, added 10.0, 5.0, 1.0, and 0.5 ppm PDMS levels significantly suppressed the thermo-oxidation of linoleic acyl groups, PUFA, and UFA, as well as further stabilising the saturated (and modified) fatty acyl groups [S(+M)FA] groups in unstirred sunflower oil (Supplementary Figure 1).

The trends observed for the nine studied PDMS concentrations in unstirred sunflower represented the rationale for focusing experiments on 10.0, 5.0, 1.0, and 0.5 ppm added PDMS concentrations in stirred sunflower oil. Similar to unstirred sunflower oil, PDMS thermo-oxidation suppressing effect of linoleic acyl groups, PUFA, and UFA acyl groups of stirred sunflower oil was also concentration-dependent with 10.0 ppm and 0.5 ppm exhibiting the maximal and minimal suppressing effects (Supplementary Figure 1). Although it has been reported that 0.05–0.06 μg cm^−2^ PDMS concentrations, which are approximately equivalent to the suppression potential of 0.025 ppm, is the minimal threshold level of PDMS required to provide a protective functional role at frying temperatures (17, 30), our ^1^H NMR investigations of the nine studied PDMS concentrations showed that 0.5 ppm was the minimum sufficient concentration, 200 centistoke (cSt) PDMS to exhibit a protective functional role in sunflower oil thermally stressed continuously at 180°C for 300 min.

#### Evolution of LOPs

Thermo-oxidation of culinary oil can be explained by the decrease in the oil unsaturated fatty acyl groups (UFA), coupled with the synchronous increase in S(+M)FA. The presence of other minor compounds in sunflower oil, as reported in section Results, could influence the thermo-oxidation susceptibility of the oil. For instance, Δ5-stigmasterol and brassicasterol exhibit free radical scavenging potential, which could suppress the formation of LOPs to a smaller extent. Physiologically, Δ7-avenasterol is a phytosterol and the chief precursor in the biosynthesis of steroids. Δ7-avenasterol has been characterised in several plant oils and has also been reported to show antioxidant activities (31, 32). Specifically, Δ7-avenasterol is reported to reduce temperature-induced oxidation of safflower oil (33).

Secondary LOPs vary widely and constitutes one or more functional group(s). By far, literature reveals alcohols, epoxides, and carbonyls, specifically, aldehydes, ketones and/or carboxylic acids to be the most reported secondary LOPs in thermo-oxidised culinary oils Martínez-Yusta et al. (23). The evolution of secondary LOPs is dependent on the oil type and their degree of unsaturation, storage and processing conditions, oxidative conditions, and the presence and type of catalyst to speed up the oxidation processes.

Primary alcohols generally undergo substitution reactions where the hydroxyl group (–O**H**) is replaced by another atom or functional group of compounds. Primary alcohols are characteristically less reactive. However, they may undergo further oxidation to form aldehydes which may also become the starting material *via* oxidation for carboxylic acids formation.

The reactivity of epoxides spans from chemically modifying nucleic acids and dimers impairing cellular functions to macro molecules reducing their digestibility (34). Specifically, 9,10-epoxy-12-octadecenoate (leukotoxin) and 12,13-Epoxy-9-octadecenoate (isoleukotoxin) stimulates proliferation of human breast cancer cells, with only leukotoxin being potent in disrupting oestrous cycle in female rats (35). Increased pulmonary vascular permeability leading to lung injury in blood-free, physiological salt solution-perfused rat lungs under constant flow conditions was reported to be caused by leukotoxin (36). Also, an intravenous injection of 100 μmol kg^−1^ leukotoxin in Wistar rats was linked to the birth of inflammatory oedema (37). The detection of epoxides in thermally stressed culinary oil means the oil may possess latent toxicity to healthy cells and organs.

Aldehydes possess a carbonyl group (>C=O), which makes it polar with a greater molecular dipole moment. The >C=O governs the chemistry of aldehydes by providing a site for nucleophilic addition and also increasing the acidity of the hydrogen atom(s) attached to the alpha carbon. Aldehydic LOPs identified and quantified in sunflower oil were either α,β-UAs or saturated aldehydes. (*E*)-2-Alkenals; (*E,E*)-2,4-alkadienals; 4,5-epoxy-(*E*)-alkenals; 4-hydroxy-(*E*)-2-alkenals; 4-hydroperoxy-(*E*)-2-alkenals; (*Z,E*)-2,4-alkadienals; (*Z*)-2-alkenals and unidentified unsaturated aldehyde (signal *k*) constituted the α,β-UAs. The saturated aldehydes generated were *n*-alkanals, 4-oxo-alkanals and low-molecular-mass *n*-alkanals such as *n*-propanal and *n*-butanal.

Unheated sunflower oil was found to contain three 0.16 ± 0.01 mM (*E*)-2-alkenals, 0.04 ± 0.00 mM (*E,E*)-2,4-alkadienals, and 0.07 ± 0.01 mM (*Z,E*)-2,4-alkadienals, as well as 0.17 ± 0.01 mM *n*-alkanals. The presence of aldehydic LOPs in unheated culinary oils may be attributed to the industrial refinement processes and/or prolonged storage conditions of the oils (1, 38, 39). The concentrations of these LOPs detectable in unheated sunflower oil may present a latent health risk to consumers.

**Figure 6:**
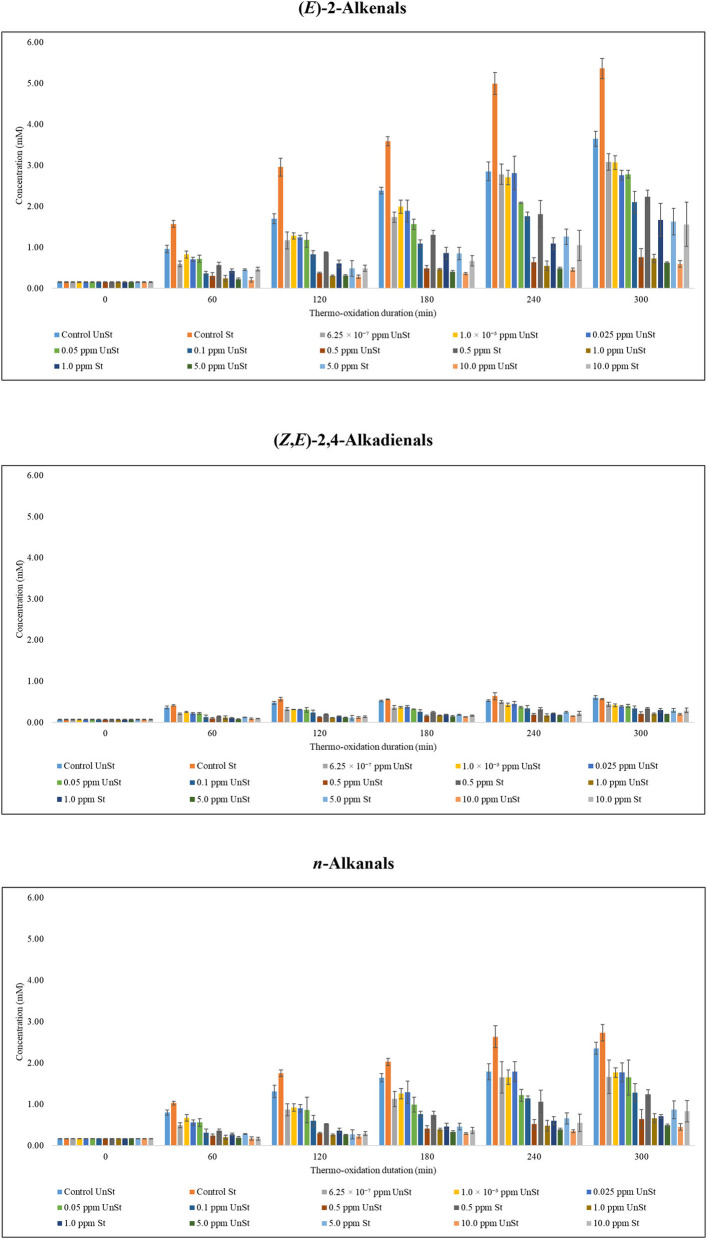
Suppression of aldehydic LOPs in PDMS-treated sunflower oil thermally stressed at 180°C continuously throughout a 300 min period.

The control experiments performed, in which sunflower oil without added PDMS were analysed, and were found to contain the highest concentrations of aldehydic LOPs (Figure 6; Supplementary Figure 2). The predominant ones were (*E*)-2-alkenals, (*E,E*)-2,4-alkadienals and *n*-alkanals. Aldehydic LOPs that were of the lowest concentrations were 4-oxo-alkanals, low-molecular-mass *n*-alkanals, (*Z*)-2-alkenals and one of the unidentified unsaturated aldehyde (signal *k*). Similar to the observations made on the acylglycerol content analysis of this oil, stirring enhanced the concentrations of LOPs detectable. In addition, 240 min was observed to be the peak time of formation of some of the aldehydic LOPs in stirred thermally stressed sunflower oil samples (Figure 6; Supplementary Figure 2).

Aldehydic LOPs in the control experiments of stirred sunflower oil was consistently higher than those in unstirred sunflower oil. On an average, they were in excess of a factor of 1.11–1.62 in stirred sunflower oil. An exception was unidentified unsaturated aldehyde (signal *k*), which was, on an average, in a 1:1 ratio of stirred sunflower oil:unstirred sunflower oil (Supplementary Figure 2). The levels of some of the aldehydic LOPs characterised in this report peaked at 240 min and decreased thereafter in stirred sunflower oil. These were (*E,E*)-2,4-alkadienals (Supplementary Figure 2); (*Z,E*)-2,4-alkadienals (Figure 6); *n*-alkanals of low molecular weight (Supplementary Figure 2); as well as unidentified unsaturated aldehyde (signal *k*) (Supplementary Figure 2). In addition, 4,5-epoxy-(*E*)-alkenals; 4-hydroperoxy-(*E*)-2-alkenals; and (*Z*)-2-alkenals levelled at 240 min with a 0.01 mM difference between 240 and 300 min of thermo-oxidation (Supplementary Figure 2). Unlike stirred sunflower oil, the concentrations of aldehydic LOPs increased in unstirred sunflower oil with respect to the oil's thermo-oxidation duration.

In a scientific report by Guillén and Uriarte (40), a volume of 4,000 mL unheated sunflower oil containing 55.05 ± 4.31% linoleoylglycerols, 34.80 ± 2.83% oleoylglycerols, 10.03 ± 0.95 SFAs, and 122.16 unit IV was submitted to a discontinuous thermo-oxidation process, with alternating room temperature storage between heating episodes and no oil replenishment in an industrial fryer (15 cm width × 30 cm length × 17 cm depth) at 190°C for an 8 h per day period for 5 days in a closed heating system. Accordingly, the total aldehyde concentrations peaked at a value of 25 mM at a thermo-oxidation duration of 2,100 min (35 h); aldehydes detectable were (*E,E*)-2,4-alkadienals, *n*-alkanals, (*E*)-2-alkenals, and (*Z,E*)-2,4-alkadienals, which peaked at 8.6, 6.6, 6.3, and 1.8 mM, respectively. In addition, 4-hydroxy-(*E*)-2-alkenals and 4-oxo-alkanals were both reported to peak at a level of 1.5 mM (40).

The data reported by Guillén and Uriarte (40) are, therefore, in agreement with the present study since the amounts of aldehydic LOPs peaked at a specified thermo-oxidation duration beyond which their amounts declined as heating progressed. It is worth noting that the lower boiling points of aldehydic LOPs and their subsequent loss from oils through evaporation at the time of thermo-oxidation of the oils may account for the decline in their levels. Furthermore, Gerde (18) reported that oxygen concentration in culinary oil increased with increased temperature until *ca*. 100°C where it abruptly decreased with increased temperature. Consequently, UFAs reacting with O_2_ was found to prevail over O_2_'s dissolution in thermally stressed oils at temperatures above 100°C (18), and this may also play a significant role in aldehydic LOPs peaking at 240 min in stirred sunflower oil in the current study.

With respect to Supplementary Equation (1), the percentage polydimethylsiloxane-suppression activity [PDMS-SA (%)] of LOPs of 6.25 × 10^−7^, 1.0 × 10^−5^, 0.025, 0.05, and 0.1 ppm treatment of unstirred sunflower oil thermally stressed continuously at 180°C were largely below 50% for a specified thermo-oxidation duration (Supplementary Table 1). Amongst the PDMS-treated unstirred sunflower oil, 0.5, 1.0, 5.0, and 10.0 ppm concentrations offered PDMS-SA values that were as low as 63%. Nonetheless, most of the PDMS-SA values for LOPs generated were consistently within the 70–85% range (Supplementary Table 1). Overall, under the same experimental conditions, 0.5, 1.0, 5.0, and 10.0 ppm PDMS-treated stirred sunflower oil samples (Supplementary Table 2) were consistently lower than their corresponding 0.5, 1.0, 5.0, and 10.0 ppm PDMS-treated unstirred sunflower oils (Supplementary Table 1), respectively.

According to Freeman et al. (30), PDMS protects against thermo-oxidation by inhibiting convective currents on a frying oil's surface, and/or by constituting an oil's surface, and in this manner produces physical layers which limit the diffusion of atmospheric O_2_ into the oil, which is a critical requirement for induction of the lipid peroxidation process. These proposed mechanisms inhibit the thermo-oxidative damage that would have been otherwise exacerbated by the reactions of atmospheric O_2_ with oil UFAs. However, agitation, be it stirring or bubbling, successfully disrupts the physical layers formed by PDMS on oil's surface, and hence the lower PDMS-SA index determined in stirred sunflower oil.

Being insoluble in oil, PDMS is also established to show a protective property by being able to form droplet suspensions in the oil. The droplet suspensions of PDMS increases their zeta potential as negatively charged species, which attracts and interacts with nearby positively charged oxygen molecules and potential low molecular weight prooxidants, thereby disrupting their kinetics and suppressing the rate of formation of LOPs (16). To prove the above, PDMS was readily dissolved at room temperature in canola oil fatty acid isopropyl ester, and this was confirmed by phase contrast microscopy (16). When thermally stressed at 180°C, dissolved PDMS exhibited no antioxidant potential in the canola oil. This was marked by tracking no observable changes in the analysed oxygen content, peroxide value, and polar compound content of the studied canola oil (16).

When comparing the same concentration for stirred and unstirred sunflower oil, aldehydic LOPs in stirred sunflower oil were, 1.20– to 2.83-fold greater than those monitored in unstirred sunflower oil (Figure 6; Supplementary Figure 2). Therefore, the presence of food of varying moisture contents and the consequential vaporisation of moisture from the fryer during cooking at high temperatures are likely to disrupt the protective layer offered by PDMS. This indicates that the PUFA-protecting potential of PDMS is likely to be from the PDMS droplets which are stably scattered throughout the oil. The high negative charge of PDMS droplets produces a suspension of charged network which reduces fluidity and, inhibit convection currents (16).

According to the United States Food and Drug Administration (41), ≤10 ppm PDMS is the legal limit allowed in food products. Exceptions include milk (0 ppm), dry gelatin dessert mixes labelled for use (≤110 ppm), ready-to-serve dry gelatin dessert (≤16 ppm), as well as salt labelled for cooking purposes (≤250 ppm), whereas ≤10 ppm is present in the cooked food (26). In addition, it has been reported that the minimum concentration of PDMS required to exhibit a protective functional role towards culinary oils thermally stressed at 180°C is 0.05–0.06 μg cm^−2^ (30).

Assuming that PDMS monomer at 180°C has a cross-sectional area of 20–25 Å^2^, all PDMS is present as an oil-air interface, and therefore the lowest concentration required to form a PDMS monolayer in oil, according to equation 1 deduced by Gerde et al. (17), is equivalent to 0.025 ppm (25 ppb). By implication, it is postulated that any PDMS concentration <0.025 ppm produces no substantial protective effect in limiting or suppressing LOPs generation in culinary oils when exposed to high-temperature frying practises (17). Data provided in the current study, however, demonstrated that 0.5 ppm was the minimal concentration of PDMS required to suppress the formation of ^1^H NMR detectable aldehydes, in both unstirred and stirred thermo-oxidised sunflower oils (Figure 6; Supplementary Figure 2).

It should also be noted that the evolution of unidentified unsaturated aldehyde (signal *k*) did not commence until the 120 min thermo-oxidation time-point in PDMS-treated unstirred sunflower oil. Similarly, (*Z*)-2-alkenals remained undetectable until the 120 min thermo-oxidation time-point continuously in 1.0, 5.0 and 10.0 ppm PDMS-treated stirred sunflower oils when heated at 180°C. However, 0.04 ± 0.01 mM (*Z*)-2-alkenals were found in 0.5 ppm PDMS- treated stirred sunflower oil that had undergone exposure to thermo-oxidation for a period of 60 min (Supplementary Figure 2). The first detection of (*Z*)-2-alkenals in 1.0 and 10.0 ppm PDMS-treated, unstirred sunflower oil was at the 240 min time-point (Supplementary Figure 2). Differences between in the evolution times of (*Z*)-2-alkenals for unstirred and stirred PDMS-treated sunflower oil samples may be attributable to disruption of the protective PDMS layers on the oil's surface, which are brought about by the stirring technique.

### Statistical Analytical Comparisons

Results acquired from an analysis of the ANOVA model depicted in Equation (2) are listed in Table 6. Overall, the added PDMS concentration source of variation was very highly significant for all aldehydes monitored, *p*-values obtained for these comparisons ranging from to 10^−131^ to 10^−64^. Further analysis involving computations of 95% confidence intervals for least square means demonstrated that even at the very low added PDMS concentration value of only 6.25 × 10^−7^ ppm, there was a significant decrease in culinary oil concentrations for all aldehydic LOPs monitored (*p* <10^−4^), with the exception of 4-hydroperoxy-(*E*)-2-alkenals, for which there was a similar level of statistical significance attained at the next higher added level (1.00 × 10^−5^ ppm). As expected, for all aldehydes, the heating time-point factor was also very highly significant (*p* <10^−63^ to 10^−137^), results clearly consistent with the propagation of the lipid peroxidation process with increasing heating periods. Likewise, the stirring effect was also statistically significant for all aldehydes except (*E,E*)-2,4-alkadienals; (*Z,E*)-2,4-alkadienals; and *n*-alkanals, with *p*-values ranging from <10^−4^ to 10^−29^, although this observation is not simply explicable. Moreover, stirring was found to give rise to elevated levels of aldehydic LOPs for all aldehydes analysed except UUA (signal *k*), for which there was an increase in its oil concentration observed in samples remaining unstirred.

**Table 6:** Statistical significance of all main sources of variation, and first-order interaction effects from ANOVA analysis of the dataset.

**Aldehyde**	**Chemical shift value (ppm)**	**(PDMS) (*P*)**	**Time-point (min) (*T*)**	**Stirring status (*S*)**	***P* × *T* interaction effect**	***P* × *S* interaction effect**	***T* × *S* interaction effect**
(*E*)-2-Alkenals	9.48	<10^−130^	<10^−131^	<10^−20^	<10^−77^	<10^−20^	<10^−7^
(*E,E*)-2,4-Alkadienals	9.52	<10^−131^	<10^−137^	ns	<10^−61^	<10^−3^	ns
4,5-Epoxy-(*E*)-2-Alkenals	9.54	<10^−130^	<10^−130^	<10^−21^	<10^−72^	3.70 × 10^−2^	<10^−8^
4-Hydroxy-(*E*)-2-Alkenals	9.56	<10^−114^	<10^−116^	<10^−29^	<10^−55^	<10^−6^	<10^−13^
4-Hydroperoxy-(*E*)-2-Alkenals	9.58	<10^−107^	<10^−111^	<10^−21^	<10^−52^	<10^−3^	<10–8
(*Z,E*)-2,4-Alkadienals	9.60	<10^−108^	<10^−104^	ns	<10^−40^	3.80 × 10^−2^	ns
*n*-Alkanals	9.74	<10^−102^	<10^−96^	ns	<10^−45^	<10^−3^	ns
4-Oxo-*n*-Alkanals	9.78	<10^−99^	<10^−115^	<10^−13^	<10^−54^	<10^−6^	<10^−5^
*n*-Alkanals (low mwt)	9.80	<10^−97^	<10^−97^	<10^−5^	<10^−31^	<10^−2^	<10^−7^
(*Z*)-2-Alkenals	10.06	<10^−107^	<10^−103^	<10^−4^	<10^−54^	ns	<10^−3^
UUA (Signal k)	10.15	<10^−106^	<10^−63^	<10^−23^	<10^−51^	ns	<10^−21^

*low mwt, low molecular weight; UUA, unidentified unsaturated aldehyde; ns, not significant*.

**Figure 7:**
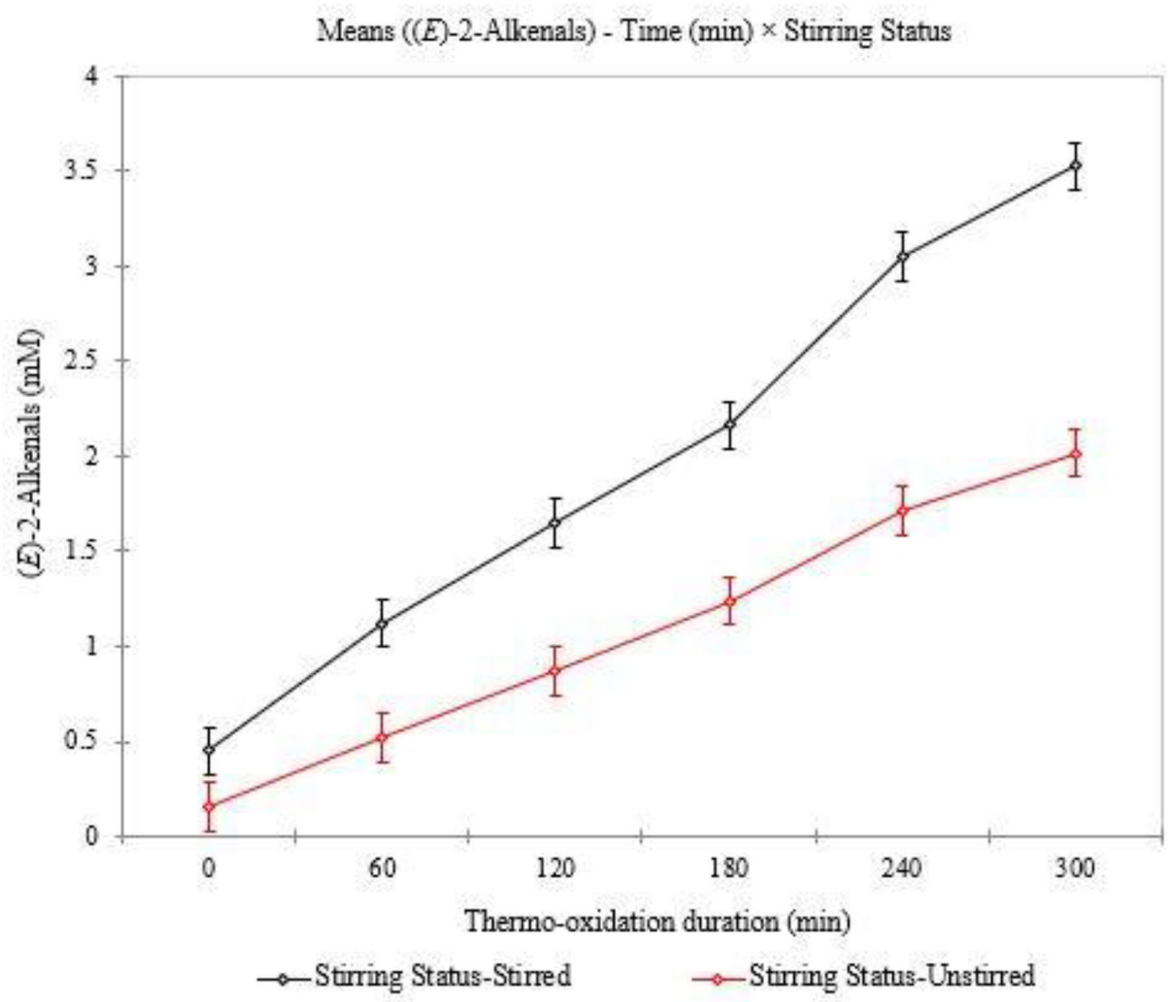
Plot of (*E*)-2-alkenals mean concentrations analysed for stirred and unstirred PDMS-treated sunflower oil.

For the first-order interaction effects investigated, the PT_*ij*_ interaction effect was significant for nearly all aldehyde variables considered (bar two), whereas those for the PS_*ik*_ effect were significant for all aldehydes except 3. The TS_*jk*_ interaction effect was significant for all aldehydes monitored. The significance of the TS_*jk*_ interaction effect can be visualised in Figure 7, where differences in the magnitudes of responses in oil (*E*)-2-alkenal concentrations are observable for samples which are stirred and unstirred.

## Conclusions

Stirring sunflower oil at 250 rpm increased the thermo-oxidation of sunflower oil's UFAs. Nonetheless, some of the aldehydic LOPs quantified in the stirred oil peaked at a time-point of 240 min, which was not the case for unstirred sunflower oil heated under the same experimental conditions. The thermo-oxidation of PDMS-treated sunflower oil showed that PDMS varied in its LOPs suppressive activity. In PDMS-unstirred sunflower oil, 6.25 × 10^−7^, 1.0 × 10^−5^, 0.025, and 0.05 ppm concentrations of this agent showed an average percentage polydimethylsiloxane-suppression activity [PDMS-SA (%)] ranging between 15.2 and 44.8%. Furthermore, at the 0.1 ppm PDMS concentration, PDMS-SA (%) varied from 40.5 to 56.3%. PDMS concentrations of 0.5, 1.0, 5.0, and 10.0 ppm either fully suppressed LOPs generation, or at least achieved a PDMS-SA (%) value of 68.4%. Comparatively, these percentages were lower in 0.5, 1.0, 5.0, and 10.0 ppm PDMS-treated, stirred sunflower oil. According to the present study's ^1^H NMR analysis findings, a 0.5 ppm PDMS concentration was found to represent the minimal threshold level of it to display a major protective functional role at the frying temperature investigated. However, the minimum added level of only 6.25 × 10^−7^ ppm was found to give rise to a statistically significant decrease in all aldehyde concentrations studied. Stirring both PDMS-free (control experiment) and PDMS-treated sunflower oils at 250 rpm also provided a foresight of the evolutionary behavioural characteristics that may be associated with the commercial, industrial, or domestic use of culinary oils exposed to high frying temperatures. It is also noteworthy that the presence of food is highly relevant, disturbing the airflow while retaining itself as part of the PDMS, potentially reducing its availability, or even altering the moisture loss pattern of the food and conditioning the fried product characteristics. Further investigations into the importance of food will be necessary for clearer understanding of PDMS behaviour in frying. Whilst it was evident that PDMS concentrations of 0.5, 1.0, 5.0, and 10.0 ppm were the most effective in suppressing LOPs generation, further synthetic and activity testing studies may be required to enhance its PDMS-SA (%) values in thermally stressed culinary oils exposed to frying temperatures, e.g., structural modification of the PDMS base molecule to incorporate an antioxidant, perhaps phenolic functional group.

## Data Availability Statement

The original contributions presented in the study are included in the article/Supplementary Material, further inquiries can be directed to the corresponding author.

## Author Contributions

DN and MG: conceptualisation and resources. AL, DN, and MG: methodology and validation. GA: formal analysis and investigation. GA and AL: data curation. AL, MG, and GA: writing—original draft preparation and visualisation. DN, MG, GA, and AL: writing—review and editing. MG: experimental design and statistical analysis. AL and DN: supervision and project administration. All authors have read and agreed to the published version of the manuscript.

## Conflict of Interest

The authors declare that the research was conducted in the absence of any commercial or financial relationships that could be construed as a potential conflict of interest.

## Publisher's Note

All claims expressed in this article are solely those of the authors and do not necessarily represent those of their affiliated organizations, or those of the publisher, the editors and the reviewers. Any product that may be evaluated in this article, or claim that may be made by its manufacturer, is not guaranteed or endorsed by the publisher.
