# Supplementary material for: The Role of Polydimethylsiloxane in Suppressing the Evolution of Lipid Oxidation Products in Thermo-Oxidised Sunflower Oil: Influence of Stirring Processes
Source: Front Nutr. 2021 Aug 10;8:721736. doi: 10.3389/fnut.2021.721736 (PMC8382684; doi:10.3389/fnut.2021.721736)
Supplement: Supplementary file 1 [file Data_Sheet_1.DOCX]

The Role of Polydimethylsiloxane in Suppressing the Evolution of Lipid Oxidation Products in Thermo-oxidised Sunflower Oil: Influence of Stirring Processes

Gilbert Ampem,^a^ Adam Le Gresley,*^a^ Martin Grootveld,^b^ and Declan P. Naughton,^a^

^a^Department of Chemistry and Pharmaceutical Sciences, SEC Faculty, Kingston University, Kingston-upon-Thames, Surrey, KT1 2EE, UK.

^b^Health and Life Sciences, De Montfort University, Leicester, LE1 9BH, UK.

^*^Corresponding author:

Dr Adam Le Gresley, Tel + 44 (0)20 84177432 Email: [a.legresley@kingston.ac.uk](mailto:a.legresley@kingston.ac.uk)

**SI.1. Discussion**

**SI.1.1. Chemical modifications to acyl groups**

The chemical modifications observed in the acyl groups of unheated sunflower oil, as well as PDMS-untreated and PDM-treated thermo-oxidised sunflower oil is shown in Figure S.1. The discussions of all observable chemical changes in the acyl groups of thermally stressed sunflower oil is provided in the manuscript under section 4.1.1.

**Oleic fatty acyl group**


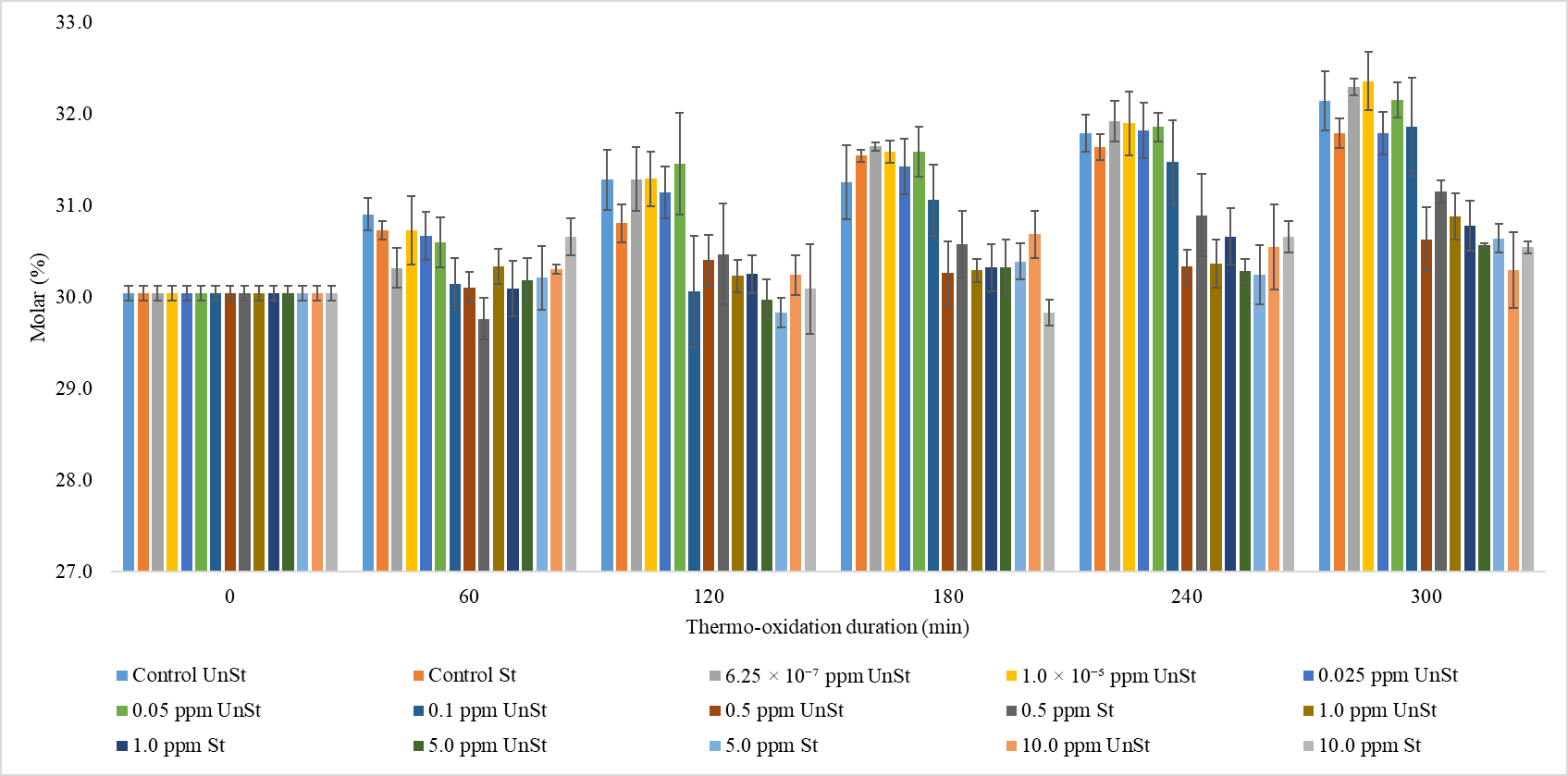

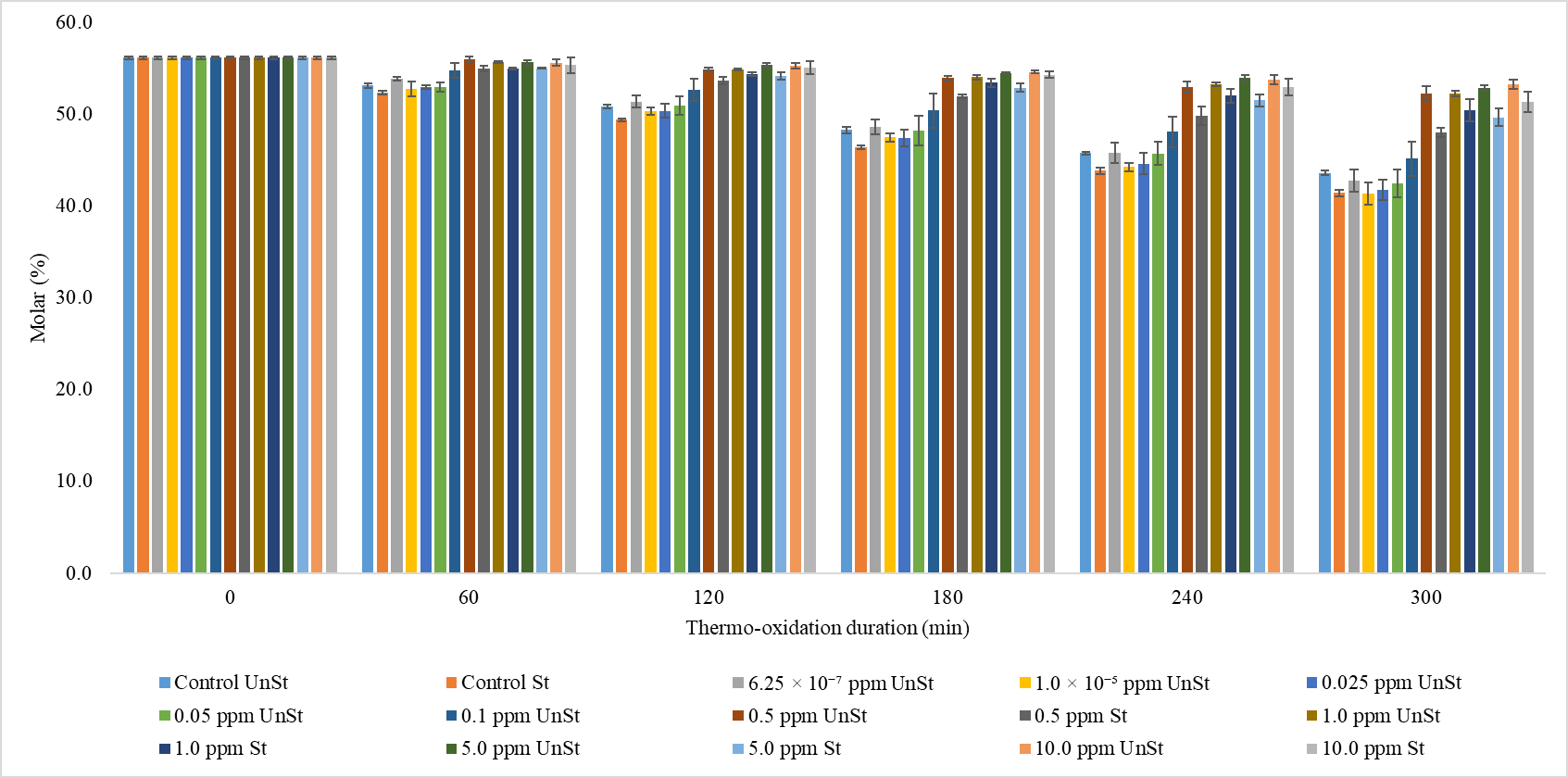


**Linoleic fatty acyl group**

**Figure S.1.** Changes in molar percentages of acyl groups and unit of iodine value of PDMS-treated sunflower oil thermally stressed at 180°C min continuously throughout a 300 period.

**Polyunsaturated acyl group**


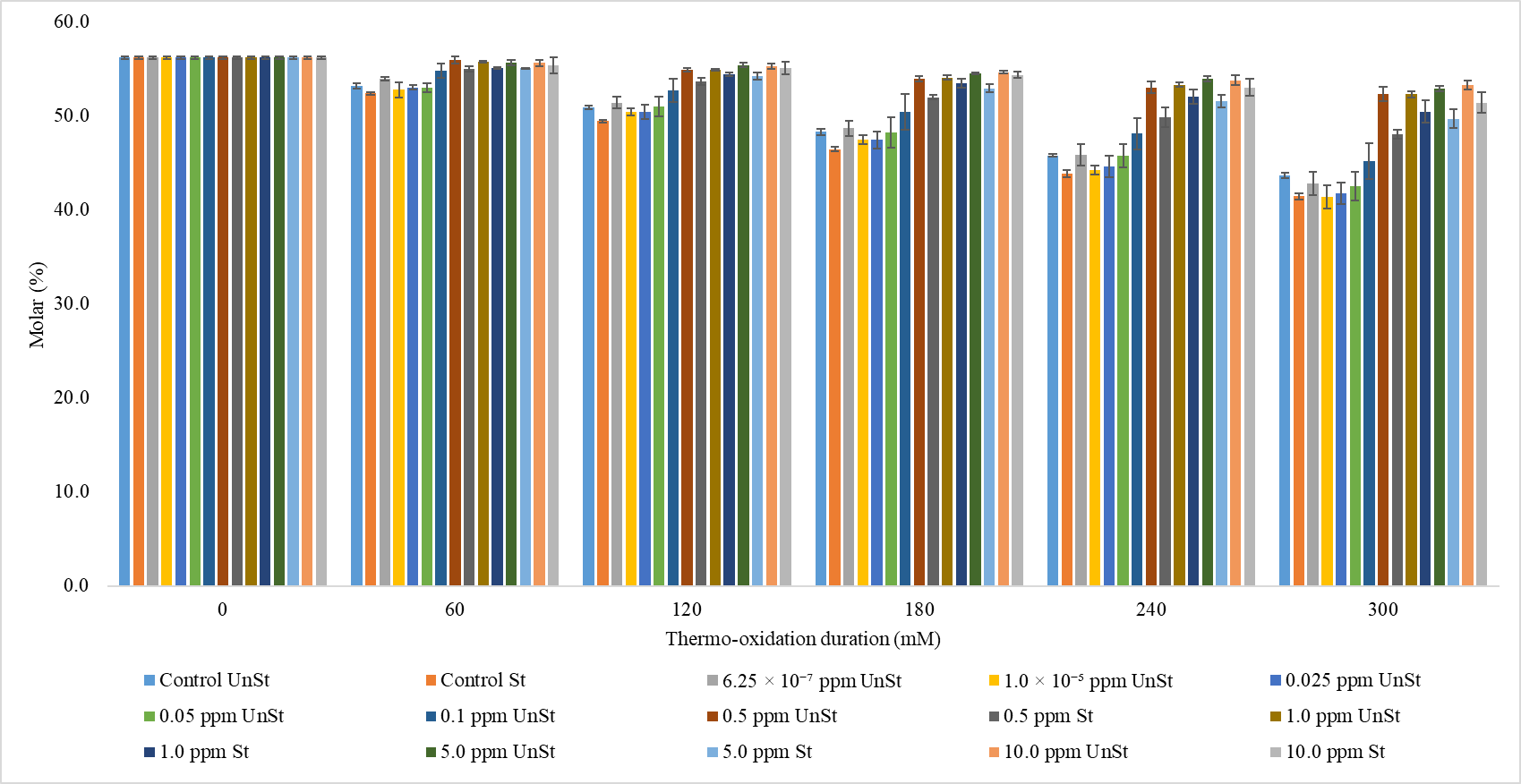


**Unsaturated acyl group**


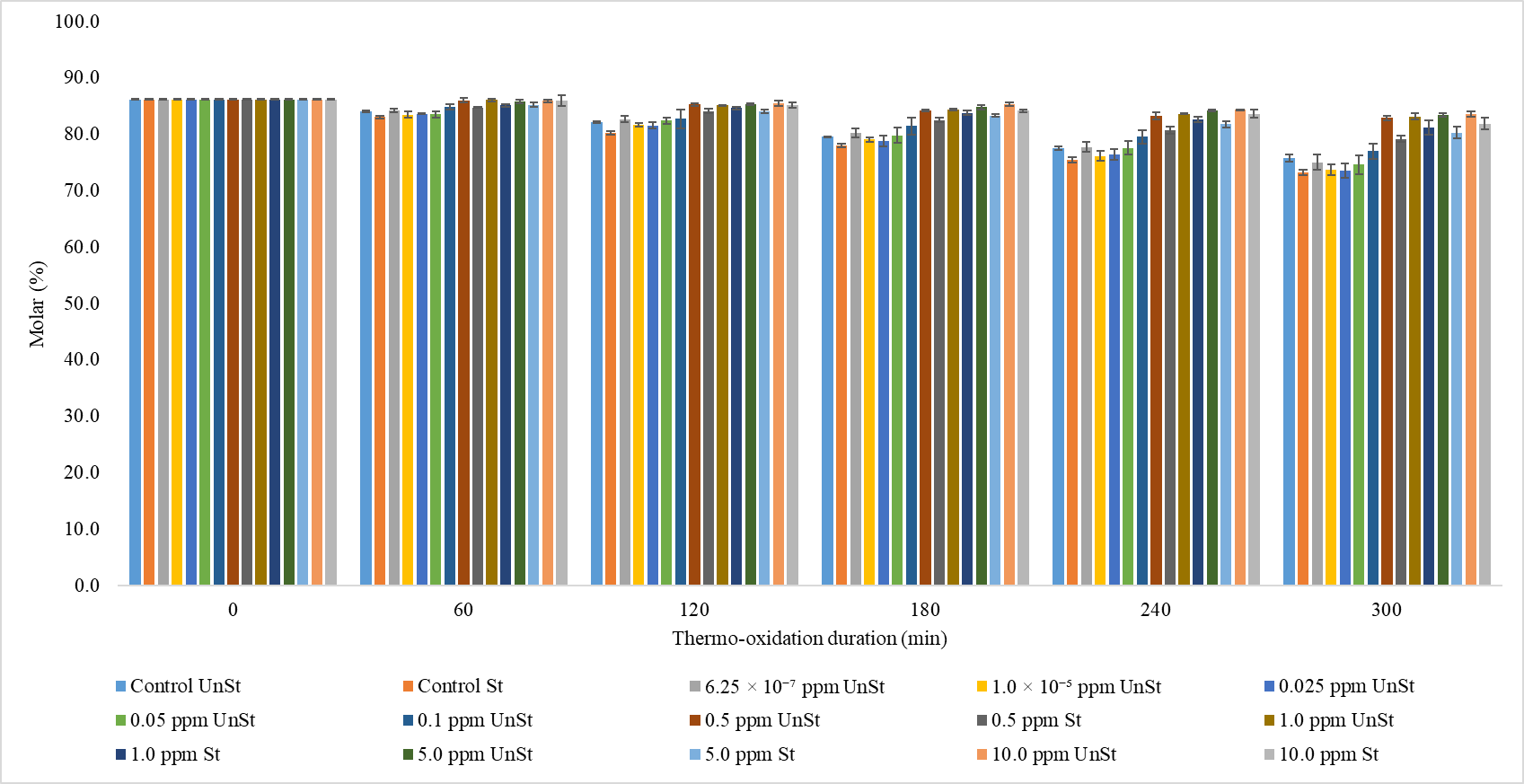


**Figure S.1.** (*Continued*) Changes in molar percentages of acyl groups and unit of iodine value of PDMS-treated sunflower oil thermally stressed at 180°C min continuously throughout a 300 period.

**Saturated (and modified) acyl groups**


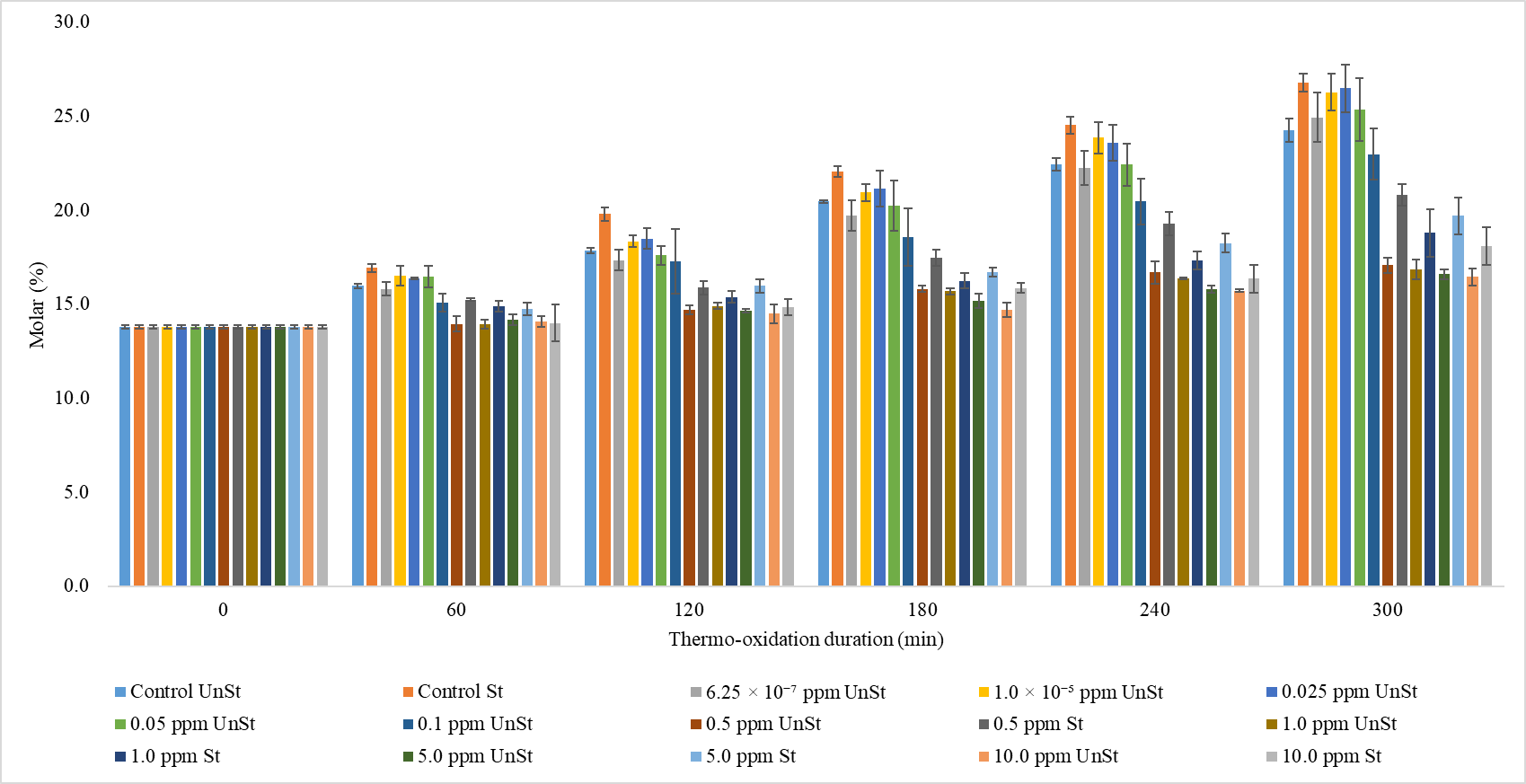

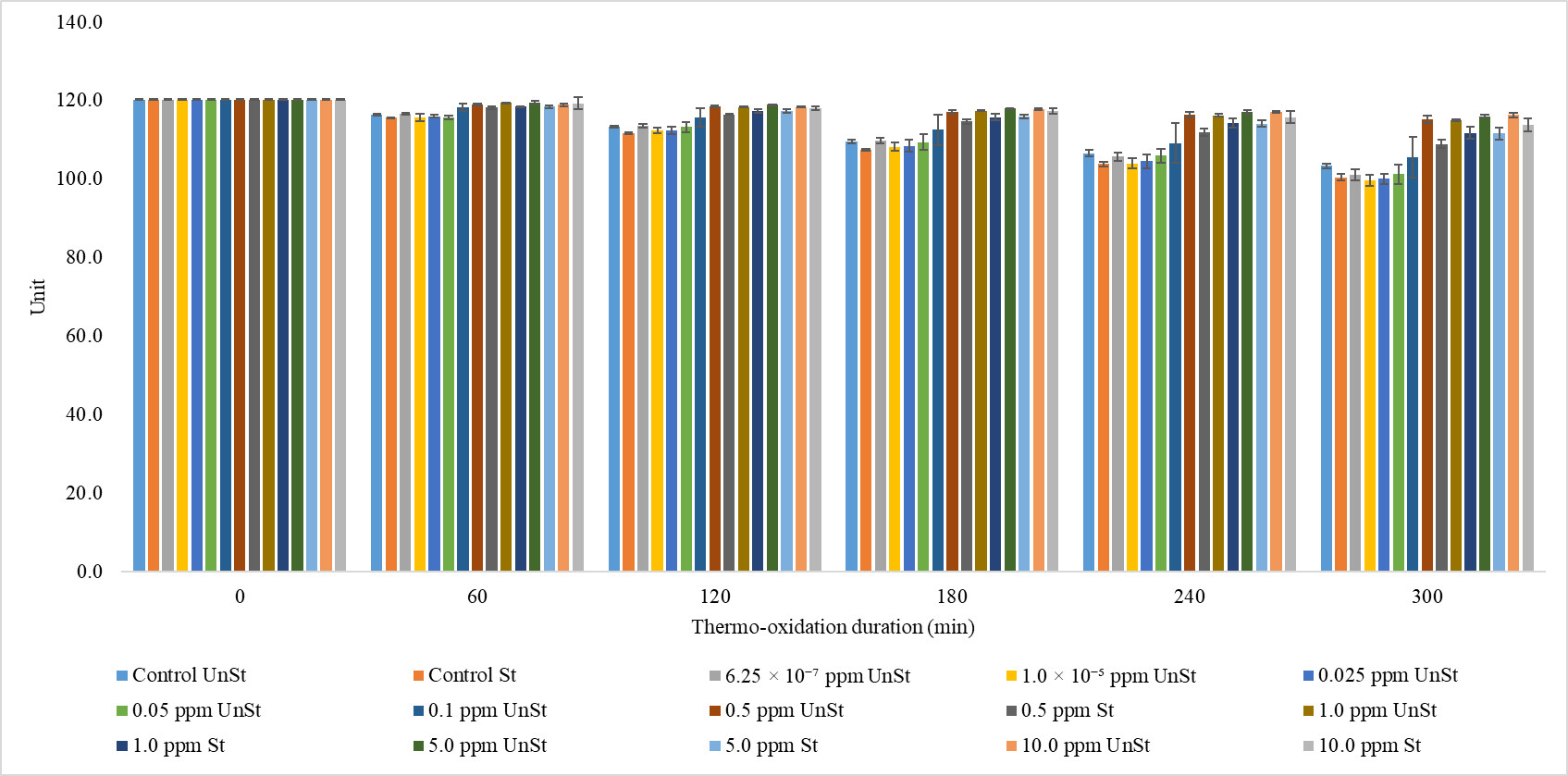


**Iodine Value**

**Figure S.1.** (*Continued*) Changes in molar percentages of acyl groups and unit of iodine value of PDMS-treated sunflower oil thermally stressed at 180°C min continuously throughout a 300 period.

**SI.1.2. Evolution of LOPs**

The chemical evolution of LOPs, as a consequence of the thermo-oxidation of the unsaturated acyl groups of unheated sunflower oil, as well as PDMS-untreated and PDM-treated thermo-oxidised sunflower oil are profiled in Figure S.2. These are also discussed in detail in the manuscript under section 4.1.2. The data profiled and presented in Figure S.2 is part of bulky aldehydic LOPs quantified with three of those namely, (*E*)-2-alkenals, (*Z*,*E*)-2,4-alkadienals, and *n*-alkanals presented in the Manuscript Figure 6.


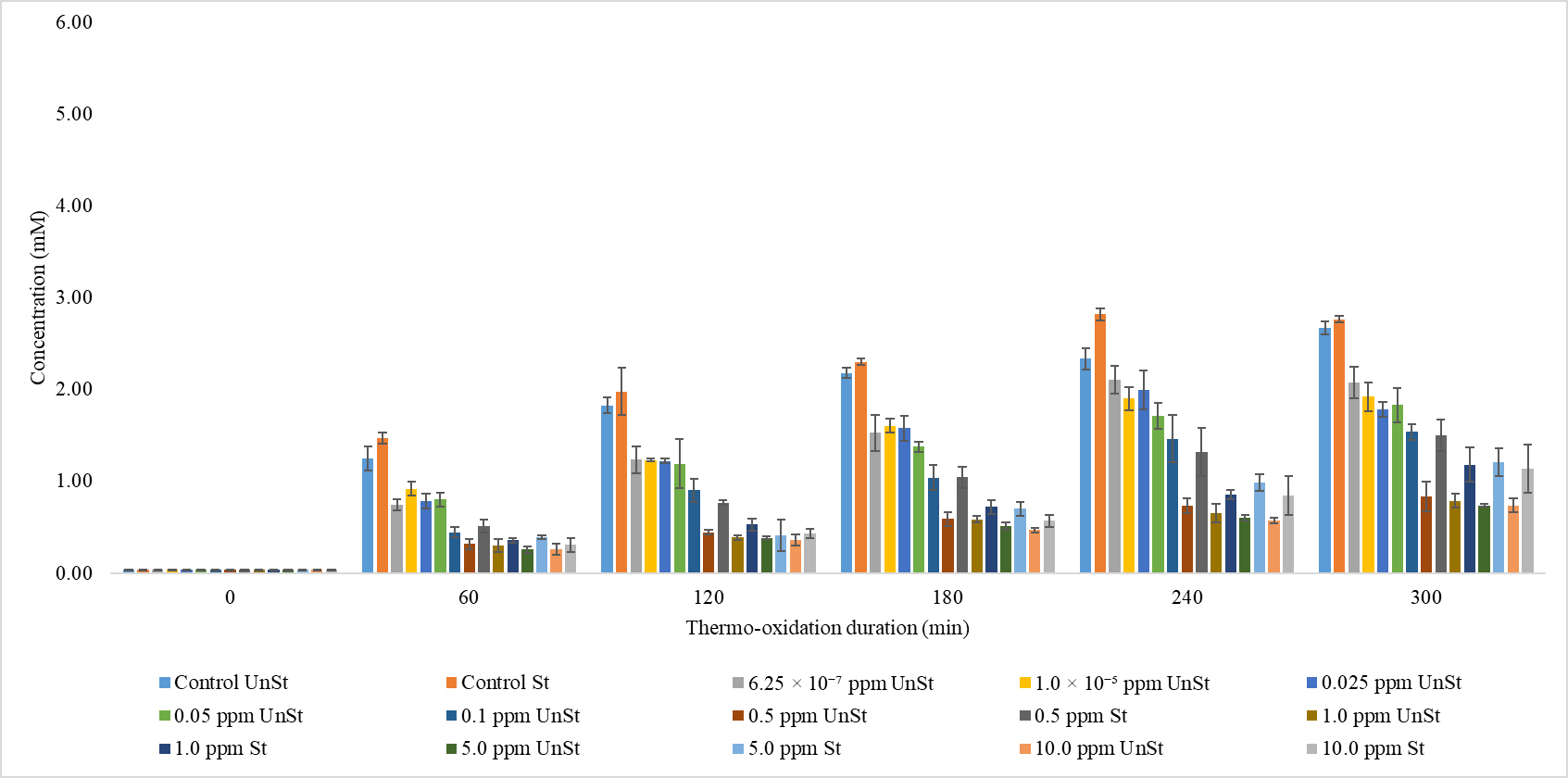


**(*E*,*E*)-2,4-Alkadienals**


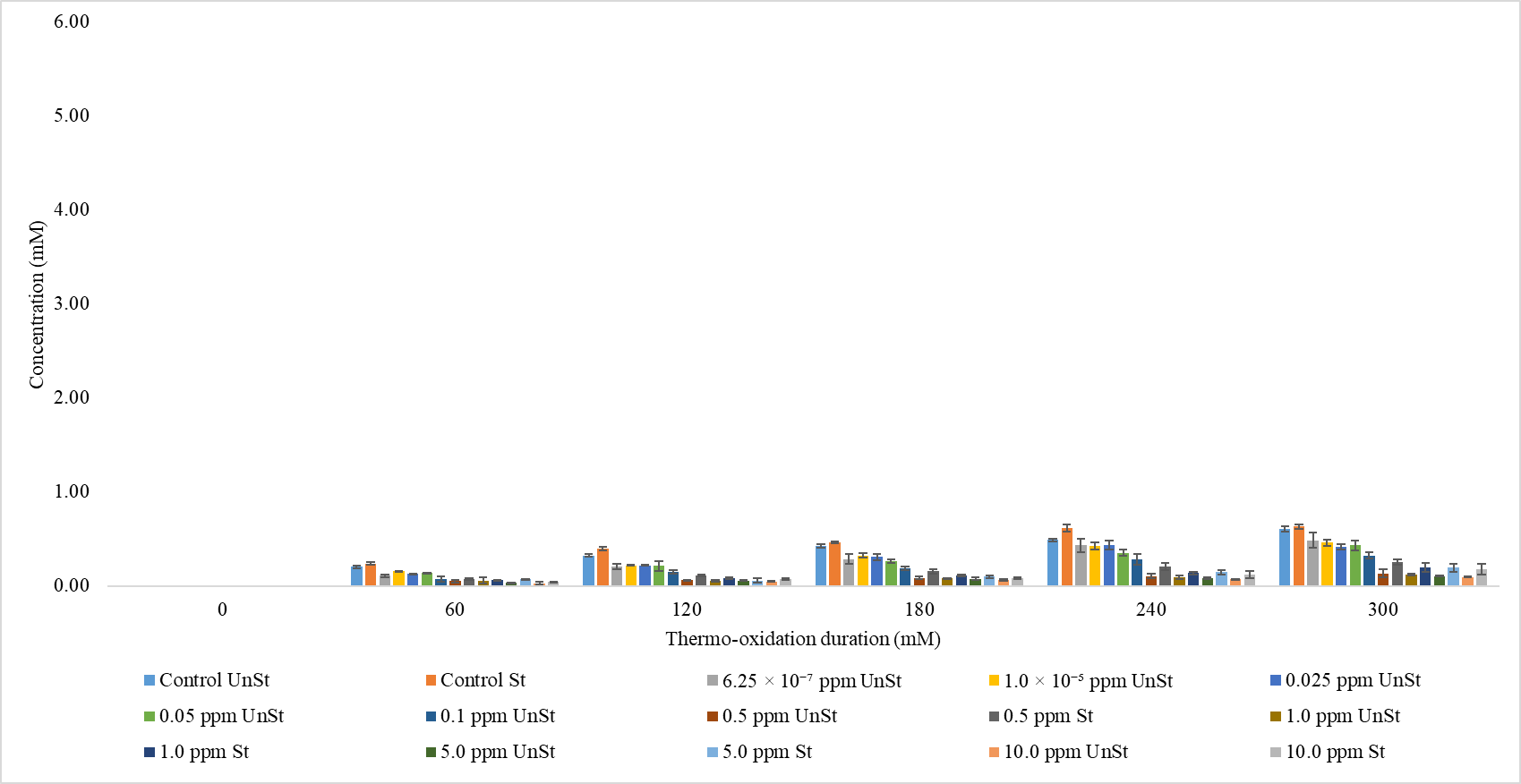


**4,5-Epoxy-(*E*)-alkenals**

**Figure S.2.** Suppression of aldehydic LOPs in PDMS-treated sunflower oil thermally stressed at 180°C min continuously throughout a 300 period.


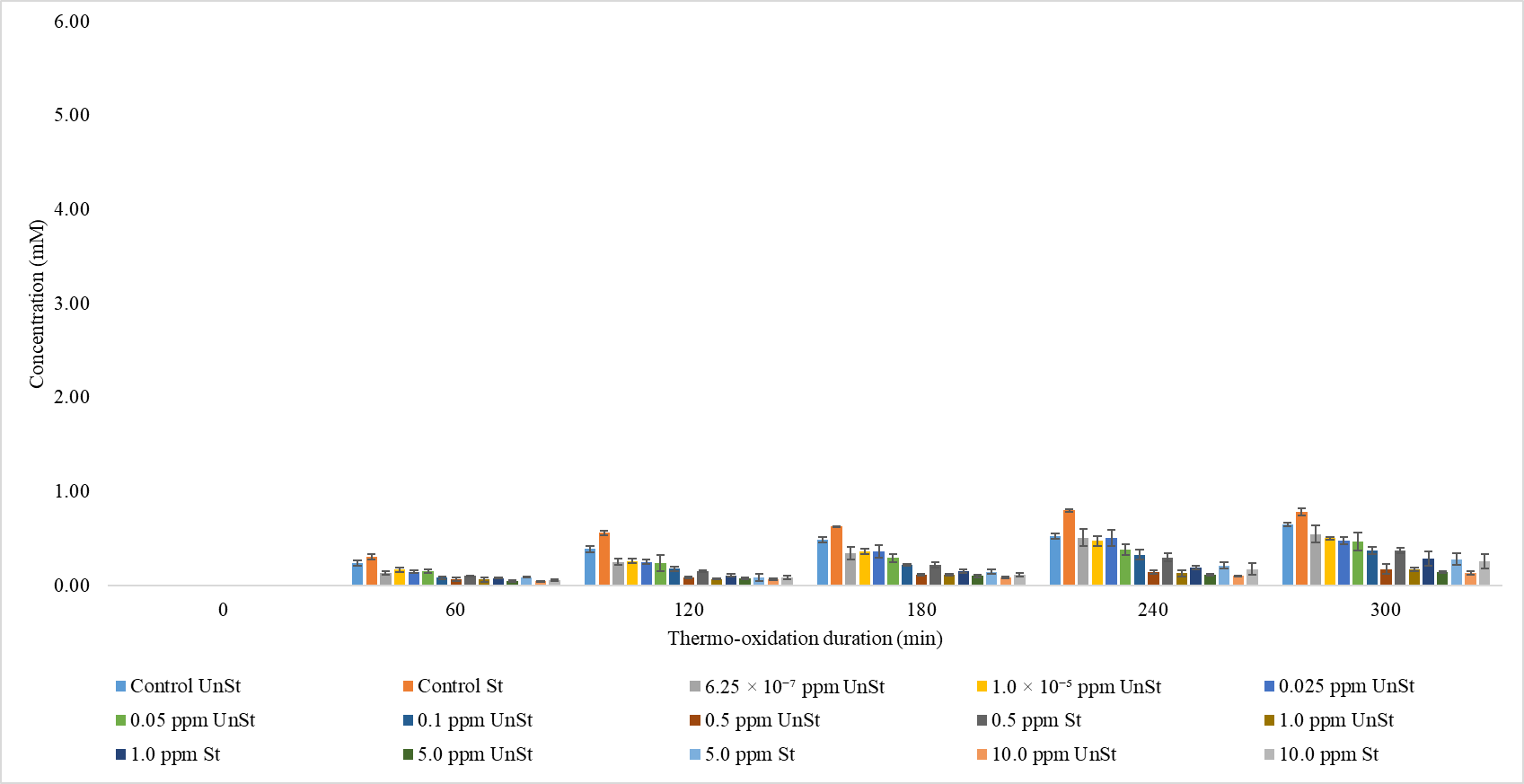


**4-Hydroxy-(*E*)-2-alkenals**


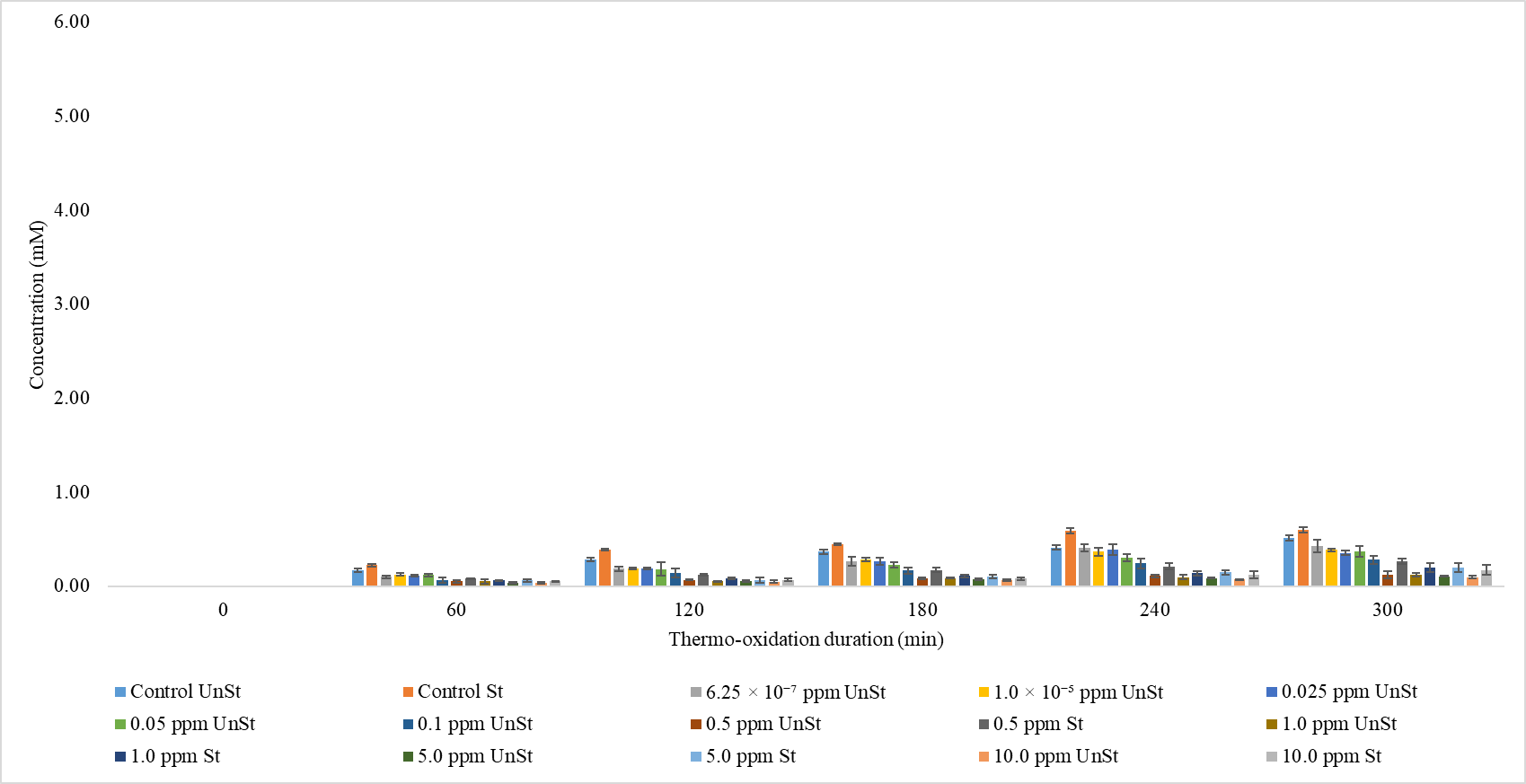


**4-Hydroperoxy-(*E*)-2-alkenals**

**Figure S.2.** (*Continued*) Suppression of aldehydic LOPs in PDMS-treated sunflower oil thermally stressed at 180°C min continuously throughout a 300 period.


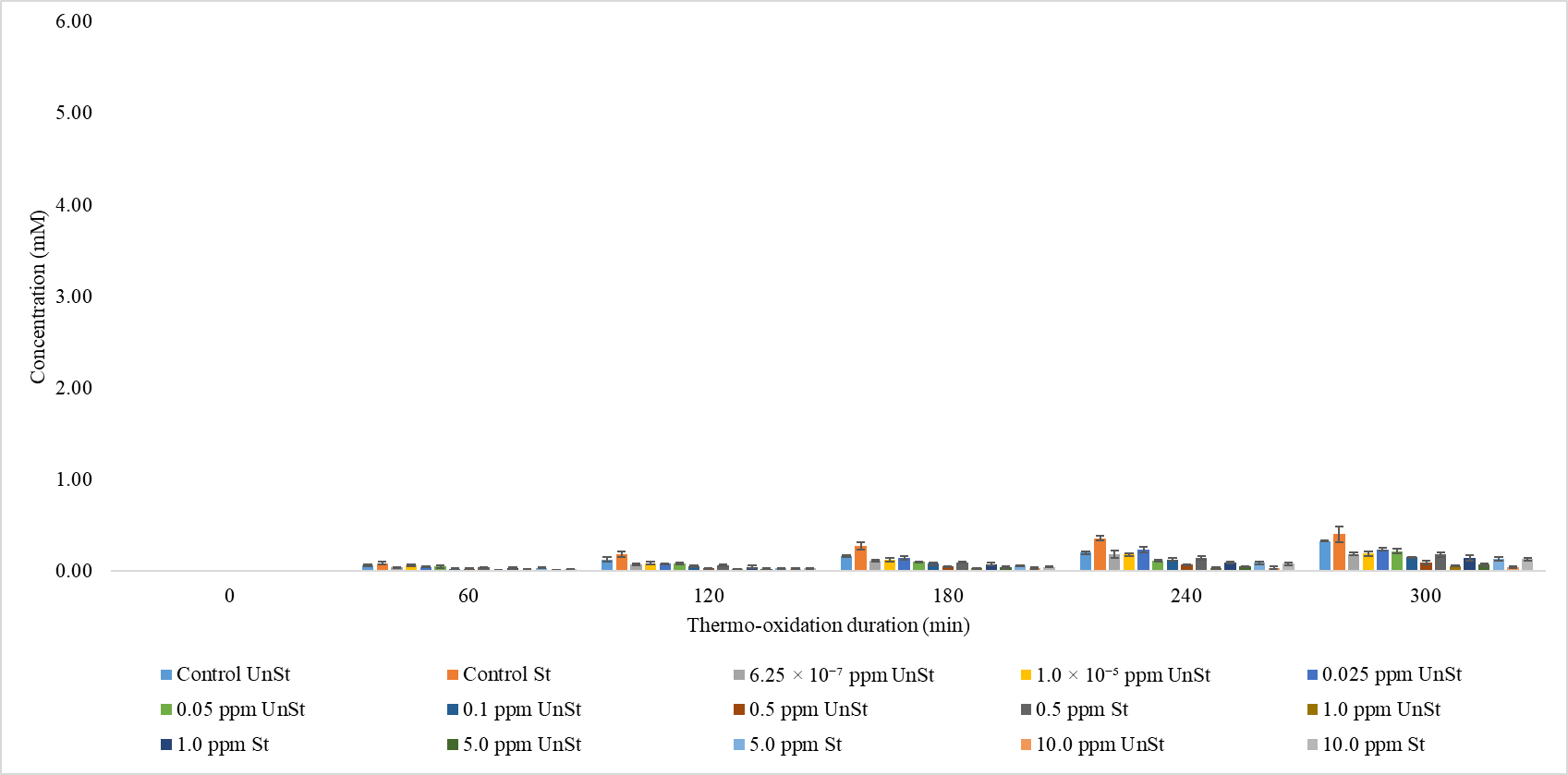


**4-Oxo-alkanals**


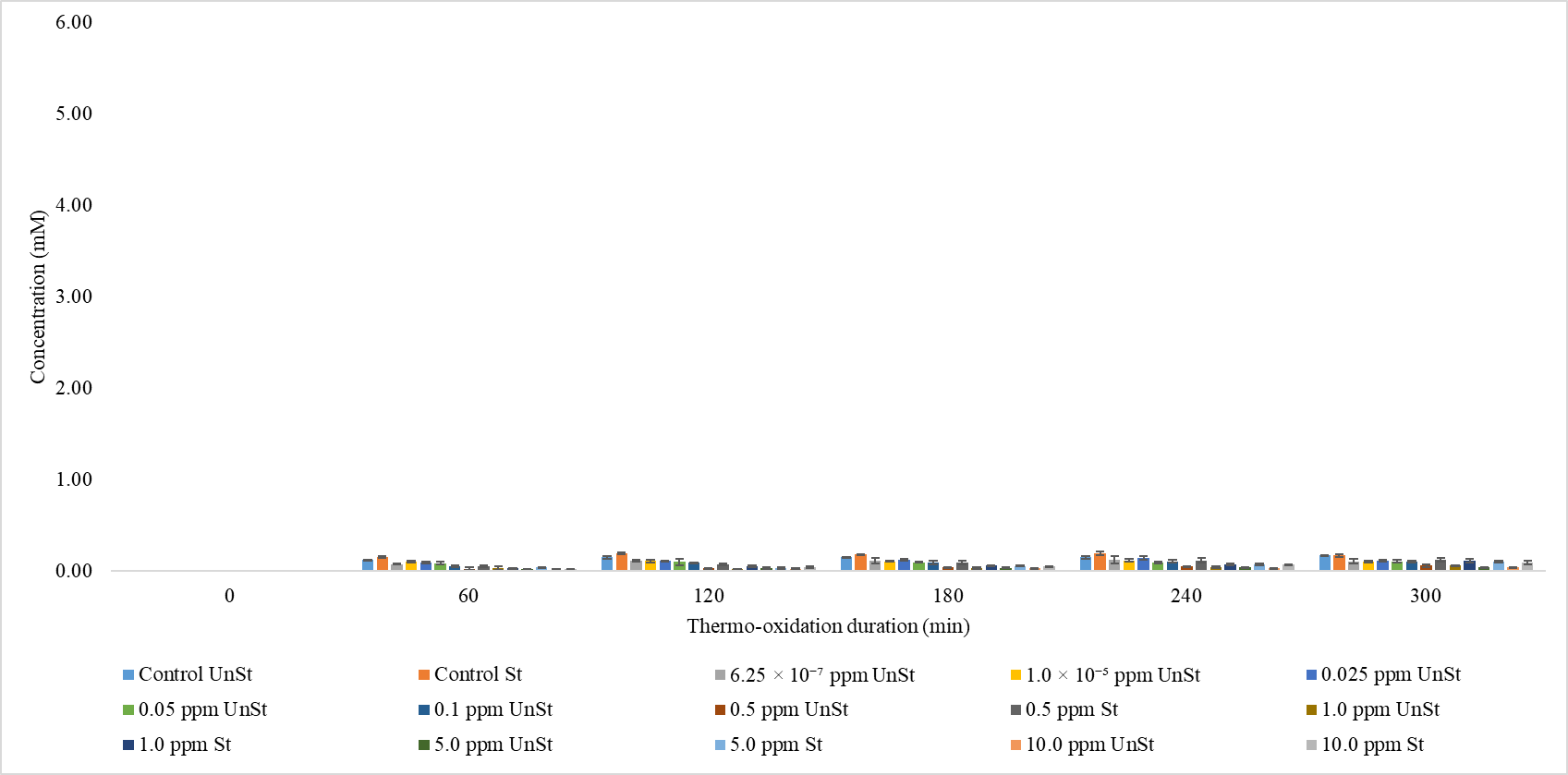


***n*-Alkanals (low molecular weight)**

**Figure S.2.** (*Continued*) Suppression of aldehydic LOPs in PDMS-treated sunflower oil thermally stressed at 180°C min continuously throughout a 300 period.


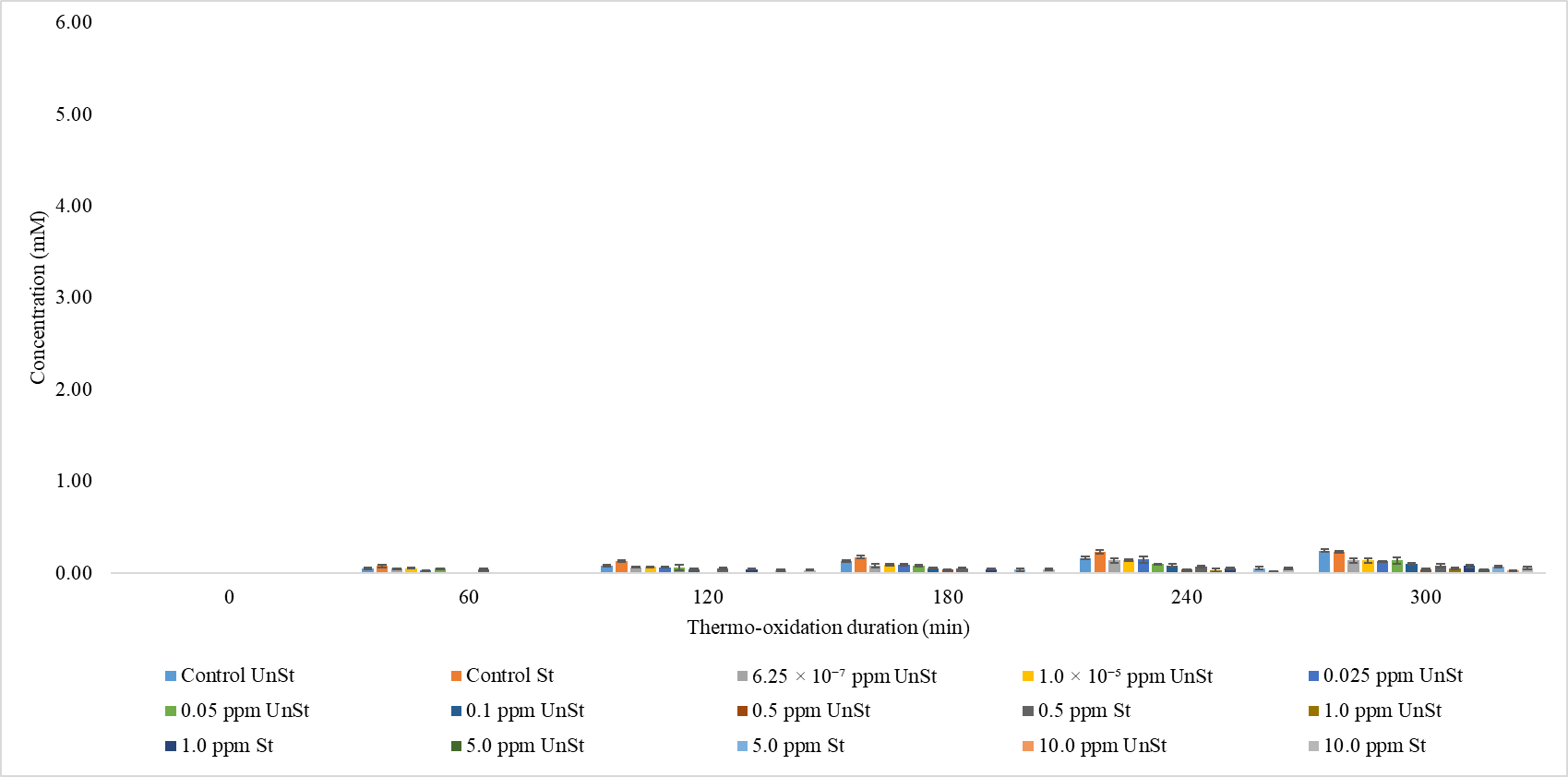


**(*Z*)-2-Alkenals**


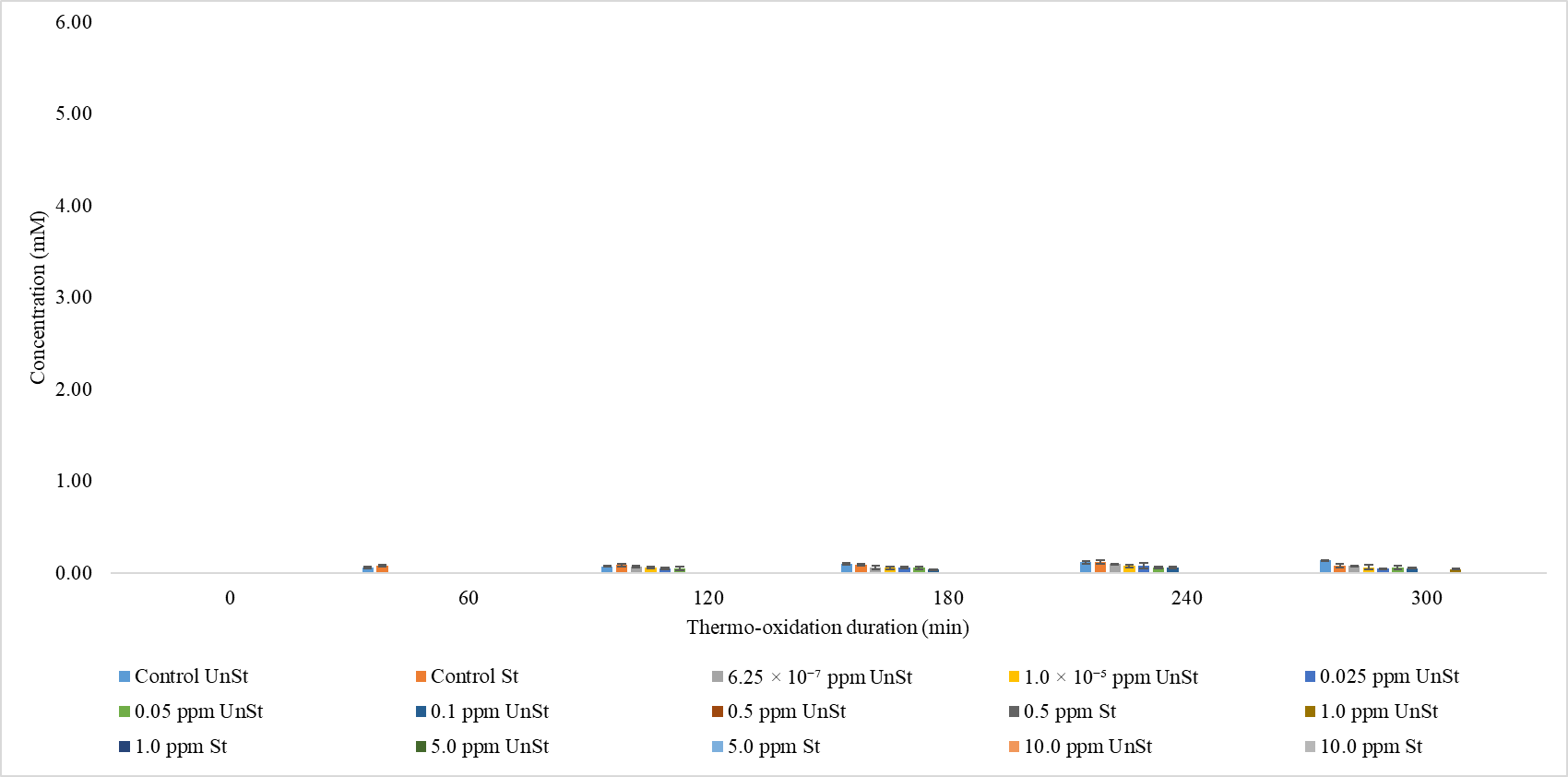


**Unidentified unsaturated aldehyde (signal k)**

**Figure S.2.** (*Continued*) Suppression of aldehydic LOPs in PDMS-treated sunflower oil thermally stressed at 180°C min continuously throughout a 300 period.

**SI.1.3. Percentage suppression activity of LOPs by PDMS**

The percentage suppression activity of a given LOPs by PDMS in PDMS-treated sunflower oil thermally stressed continuously at 180°C for a specified thermo-oxidative time period was generated by equation S1.

PDMS-SA (%) = 100^a^ – ($\frac{LOPs quantified in PDMS ̵treated sunflower oil (mM)}{LOPs quantified in contol experiemnt (mM)})$ × 100^b^ (S1)

PDMS-SA (%) is the percentage polydimethylsiloxane-suppression activity of LOPs. The assumption is that the concentration of a given LOPs in mM in the PDMS-free sunflower oil (control experiment) is always equivalent to 100% hence, 100^a^. This is only applicable to a specified heating duration. As a result, the comparison of the varying concentrations of PDMS treatment in Table 6 and 7 in the manuscript reads across the table and is only limited to a specified heating duration of the studied culinary oil. The 100^b^ is the percentage conversion.

**Table S.1.** Percentage suppression activity of LOPs by PDMS in PDMS-treated unstirred sunflower oil.

|  |  |  | Percentage suppression activity of LOPs by PDMS treatment | | | | | | | | |
| --- | --- | --- | --- | --- | --- | --- | --- | --- | --- | --- | --- |
| Thermo-oxidation duration (min) | Control (Unstirred) (mM) | Control (Unstirred) (% equivalence) | 6.25 × 10⁻⁷ ppm | 1.0 × 10⁻⁵ ppm | 0.025 ppm | 0.05 ppm | 0.1 ppm | 0.5 ppm | 1.0 ppm | 5.0 ppm | 10.0 ppm |
| (*E*)-2-Alkenals | | | | | | | | | | | |
| 0 (Unheated) | 0.16 | 100 | n/a | n/a | n/a | n/a | n/a | n/a | n/a | n/a | n/a |
| 60 | 0.96 | 100 | 37.49 | 13.93 | 26.48 | 24.75 | 61.43 | 68.00 | 74.11 | 76.21 | 78.44 |
| 120 | 1.70 | 100 | 30.93 | 24.58 | 26.96 | 30.48 | 50.96 | 77.58 | 81.87 | 81.24 | 83.07 |
| 180 | 2.39 | 100 | 27.30 | 16.58 | 20.89 | 34.44 | 54.22 | 79.35 | 80.40 | 82.77 | 84.70 |
| 240 | 2.85 | 100 | 2.48 | 5.17 | 1.47 | 26.84 | 38.22 | 77.57 | 80.66 | 82.78 | 84.04 |
| 300 | 3.65 | 100 | 15.49 | 15.92 | 24.53 | 23.72 | 42.42 | 79.16 | 80.05 | 82.87 | 83.53 |
| Average | 1.95 | 100 | 22.74 | 15.24 | 20.07 | 28.05 | 49.45 | 76.33 | 79.42 | 81.17 | 82.75 |
|  | | | | | | | | | | | |
| (*E*,*E*)-2,4-Alkadienals | | | | | | | | | | | |
| 0 (Unheated) | 0.04 | 100 | n/a | n/a | n/a | n/a | n/a | n/a | n/a | n/a | n/a |
| 60 | 1.25 | 100 | 40.51 | 26.44 | 37.30 | 35.83 | 64.33 | 74.64 | 75.74 | 79.54 | 79.28 |
| 120 | 1.83 | 100 | 32.42 | 32.59 | 33.08 | 34.70 | 50.67 | 75.60 | 78.83 | 78.92 | 80.20 |
| 180 | 2.18 | 100 | 29.89 | 26.48 | 27.65 | 36.94 | 52.40 | 72.93 | 73.18 | 76.62 | 78.49 |
| 240 | 2.34 | 100 | 9.74 | 18.62 | 14.58 | 26.71 | 37.23 | 68.42 | 71.90 | 74.10 | 75.43 |
| 300 | 2.67 | 100 | 22.33 | 28.13 | 33.26 | 31.51 | 42.48 | 68.85 | 70.51 | 72.48 | 72.44 |
| Average | 1.72 | 100 | 26.98 | 26.45 | 29.18 | 33.14 | 49.42 | 72.09 | 74.03 | 76.33 | 77.17 |

Across the table, percentage suppression activity of LOPs by PDMS for each PDMS concentration is with respect to the LOPs concentration measured in the control (unstirred) sunflower oil thermally stressed at the corresponding thermo-oxidation duration (min). Not applicable (n/a) implies no measurement at the corresponding thermo-oxidation duration (min).

**Table S.1.** (*Continued*) Percentage suppression activity of LOPs by PDMS in PDMS-treated unstirred sunflower oil.

|  |  |  | Percentage suppression activity of LOPs by PDMS treatment | | | | | | | | |
| --- | --- | --- | --- | --- | --- | --- | --- | --- | --- | --- | --- |
| Thermo-oxidation duration (min) | Control (Unstirred) (mM) | Control (Unstirred) (% equivalence) | 6.25 × 10⁻⁷ ppm | 1.0 × 10⁻⁵ ppm | 0.025 ppm | 0.05 ppm | 0.1 ppm | 0.5 ppm | 1.0 ppm | 5.0 ppm | 10.0 ppm |
| 4,5-Epoxy-(*E*)-alkenals | | | | | | | | | | | |
| 0 (Unheated) | - | - | - | - | - | - | - | - | - | - | - |
| 60 | 0.20 | 100 | 47.21 | 23.45 | 36.83 | 32.57 | 65.58 | 75.26 | 71.13 | 82.15 | 85.01 |
| 120 | 0.33 | 100 | 35.75 | 32.41 | 32.28 | 34.59 | 54.23 | 80.44 | 83.84 | 82.13 | 84.70 |
| 180 | 0.43 | 100 | 32.82 | 24.29 | 28.06 | 38.32 | 56.18 | 79.76 | 79.94 | 82.02 | 85.25 |
| 240 | 0.49 | 100 | 11.85 | 13.40 | 11.01 | 27.64 | 42.42 | 78.22 | 80.71 | 82.76 | 85.22 |
| 300 | 0.60 | 100 | 19.52 | 23.86 | 31.69 | 28.63 | 46.42 | 78.07 | 79.62 | 82.47 | 84.05 |
| Average | 0.34 | 100 | 29.43 | 23.48 | 27.97 | 32.35 | 52.97 | 78.35 | 79.05 | 82.30 | 84.85 |
|  | | | | | | | | | | | |
| 4-Hydroxy-(*E*)-2-alkenals | | | | | | | | | | | |
| 0 (Unheated) | - | - | - | - | - | - | - | - | - | - | - |
| 60 | 0.24 | 100 | 44.25 | 30.39 | 39.07 | 37.79 | 66.01 | 72.11 | 74.33 | 79.61 | 82.50 |
| 120 | 0.39 | 100 | 34.88 | 33.04 | 35.16 | 38.16 | 54.44 | 77.53 | 82.46 | 80.28 | 83.28 |
| 180 | 0.49 | 100 | 29.73 | 25.16 | 25.86 | 40.28 | 55.33 | 76.28 | 77.07 | 79.06 | 83.20 |
| 240 | 0.53 | 100 | 3.82 | 10.38 | 4.22 | 27.57 | 37.96 | 72.93 | 75.67 | 77.92 | 80.97 |
| 300 | 0.65 | 100 | 15.84 | 23.00 | 27.12 | 28.34 | 43.01 | 73.11 | 74.39 | 77.78 | 79.57 |
| Average | 0.38 | 100 | 25.70 | 24.39 | 26.29 | 34.43 | 51.35 | 74.39 | 76.79 | 78.93 | 81.90 |

Across the table, percentage suppression activity of LOPs by PDMS for each PDMS concentration is with respect to the LOPs concentration measured in the control (unstirred) sunflower oil thermally stressed at the corresponding thermo-oxidation duration (min). Not applicable (n/a) implies no measurement at the corresponding thermo-oxidation duration (min).

**Table S.1.** (*Continued*) Percentage suppression activity of LOPs by PDMS in PDMS-treated unstirred sunflower oil.

|  |  |  | Percentage suppression activity of LOPs by PDMS treatment | | | | | | | | |
| --- | --- | --- | --- | --- | --- | --- | --- | --- | --- | --- | --- |
| Thermo-oxidation duration (min) | Control (Unstirred) (mM) | Control (Unstirred) (% equivalence) | 6.25 × 10⁻⁷ ppm | 1.0 × 10⁻⁵ ppm | 0.025 ppm | 0.05 ppm | 0.1 ppm | 0.5 ppm | 1.0 ppm | 5.0 ppm | 10.0 ppm |
| 4-Hydroperoxy-(*E*)-2-alkenals | | | | | | | | | | | |
| 0 (Unheated) | - | - | - | - | - | - | - | - | - | - | - |
| 60 | 0.17 | 100 | 43.12 | 28.09 | 37.31 | 31.85 | 63.01 | 70.55 | 71.34 | 78.62 | 80.82 |
| 120 | 0.28 | 100 | 34.23 | 33.19 | 34.61 | 35.64 | 50.95 | 77.27 | 81.71 | 80.47 | 82.70 |
| 180 | 0.37 | 100 | 27.62 | 23.17 | 28.63 | 38.10 | 55.34 | 77.39 | 77.33 | 79.98 | 83.21 |
| 240 | 0.41 | 100 | 1.45 | 11.11 | 5.68 | 26.72 | 41.28 | 73.77 | 76.67 | 79.62 | 82.45 |
| 300 | 0.51 | 100 | 17.48 | 24.73 | 30.93 | 28.54 | 45.41 | 76.43 | 76.39 | 79.71 | 81.59 |
| Average | 0.29 | 100 | 24.78 | 24.06 | 27.43 | 32.17 | 51.20 | 75.08 | 76.69 | 79.68 | 82.15 |
|  | | | | | | | | | | | |
| (*Z*,*E*)-2,4-Alkadienals | | | | | | | | | | | |
| 0 (Unheated) | 0.07 | 100 | n/a | n/a | n/a | n/a | n/a | n/a | n/a | n/a | n/a |
| 60 | 0.36 | 100 | 41.77 | 30.03 | 40.86 | 39.81 | 64.82 | 72.93 | 66.48 | 77.16 | 76.11 |
| 120 | 0.48 | 100 | 31.99 | 33.33 | 34.72 | 36.02 | 50.33 | 72.34 | 75.29 | 74.56 | 75.94 |
| 180 | 0.52 | 100 | 30.52 | 29.15 | 27.27 | 38.37 | 49.35 | 68.75 | 67.54 | 70.85 | 74.25 |
| 240 | 0.53 | 100 | 6.54 | 19.00 | 14.47 | 30.24 | 35.24 | 63.47 | 67.25 | 67.95 | 70.79 |
| 300 | 0.61 | 100 | 26.65 | 31.22 | 35.74 | 33.80 | 43.12 | 64.62 | 66.16 | 67.84 | 66.77 |
| Average | 0.43 | 100 | 27.49 | 28.55 | 30.61 | 35.65 | 48.57 | 68.42 | 68.54 | 71.67 | 72.77 |

Across the table, percentage suppression activity of LOPs by PDMS for each PDMS concentration is with respect to the LOPs concentration measured in the control (unstirred) sunflower oil thermally stressed at the corresponding thermo-oxidation duration (min). Not applicable (n/a) implies no measurement at the corresponding thermo-oxidation duration (min).

**Table S.1.** (*Continued*) Percentage suppression activity of LOPs by PDMS in PDMS-treated unstirred sunflower oil.

|  |  |  | Percentage suppression activity of LOPs by PDMS treatment | | | | | | | | |
| --- | --- | --- | --- | --- | --- | --- | --- | --- | --- | --- | --- |
| Thermo-oxidation duration (min) | Control (Unstirred) (mM) | Control (Unstirred) (% equivalence) | 6.25 × 10⁻⁷ ppm | 1.0 × 10⁻⁵ ppm | 0.025 ppm | 0.05 ppm | 0.1 ppm | 0.5 ppm | 1.0 ppm | 5.0 ppm | 10.0 ppm |
| *n*-Alkanals | | | | | | | | | | | |
| 0 (Unheated) | 0.17 | 100 | n/a | n/a | n/a | n/a | n/a | n/a | n/a | n/a | n/a |
| 60 | 0.80 | 100 | 38.49 | 16.27 | 30.23 | 30.07 | 61.32 | 69.64 | 74.33 | 75.92 | 78.03 |
| 120 | 1.31 | 100 | 33.72 | 29.60 | 31.05 | 34.53 | 54.00 | 77.01 | 80.17 | 80.09 | 82.77 |
| 180 | 1.64 | 100 | 31.11 | 23.25 | 21.25 | 39.51 | 53.42 | 75.10 | 76.06 | 79.91 | 82.20 |
| 240 | 1.79 | 100 | 7.47 | 7.39 | -0.20 | 31.81 | 36.04 | 70.92 | 73.26 | 78.14 | 80.33 |
| 300 | 2.36 | 100 | 29.34 | 24.94 | 24.80 | 29.98 | 45.61 | 72.70 | 71.88 | 79.08 | 80.74 |
| Average | 1.35 | 100 | 28.03 | 20.29 | 21.42 | 33.18 | 50.08 | 73.07 | 75.14 | 78.63 | 80.81 |
|  | | | | | | | | | | | |
| 4-Oxo-alkanals | | | | | | | | | | | |
| 0 (Unheated) | - | - | - | - | - | - | - | - | - | - | - |
| 60 | 0.06 | 100 | 40.55 | 7.55 | 29.47 | 23.91 | 64.45 | 65.64 | 74.12 | 74.56 | 79.44 |
| 120 | 0.13 | 100 | 45.48 | 34.03 | 35.73 | 35.09 | 59.68 | 78.15 | 86.06 | 80.72 | 80.52 |
| 180 | 0.16 | 100 | 33.47 | 26.65 | 12.88 | 39.41 | 51.12 | 71.02 | 83.56 | 75.50 | 80.48 |
| 240 | 0.20 | 100 | 10.39 | 10.23 | -15.96 | 42.28 | 36.33 | 64.86 | 82.39 | 74.89 | 81.74 |
| 300 | 0.33 | 100 | 42.90 | 41.81 | 27.83 | 33.49 | 53.49 | 71.14 | 83.24 | 78.42 | 86.47 |
| Average | 0.15 | 100 | 34.56 | 24.05 | 17.99 | 34.84 | 53.01 | 70.16 | 81.87 | 76.82 | 81.73 |

Across the table, percentage suppression activity of LOPs by PDMS for each PDMS concentration is with respect to the LOPs concentration measured in the control (unstirred) sunflower oil thermally stressed at the corresponding thermo-oxidation duration (min). Not applicable (n/a) implies no measurement at the corresponding thermo-oxidation duration (min).

**Table S.1.** (*Continued*) Percentage suppression activity of LOPs by PDMS in PDMS-treated unstirred sunflower oil.

|  |  |  | Percentage suppression activity of LOPs by PDMS treatment | | | | | | | | |
| --- | --- | --- | --- | --- | --- | --- | --- | --- | --- | --- | --- |
| Thermo-oxidation duration (min) | Control (Unstirred) (mM) | Control (Unstirred) (% equivalence) | 6.25 × 10⁻⁷ ppm | 1.0 × 10⁻⁵ ppm | 0.025 ppm | 0.05 ppm | 0.1 ppm | 0.5 ppm | 1.0 ppm | 5.0 ppm | 10.0 ppm |
| *n*-Alkanals (low molecular weight) | | | | | | | | | | | |
| 0 (Unheated) | - | - | - | - | - | - | - | - | - | - | - |
| 60 | 0.12 | 100 | 36.61 | 14.27 | 24.80 | 28.39 | 55.10 | 77.06 | 70.33 | 81.18 | 84.92 |
| 120 | 0.15 | 100 | 24.57 | 26.59 | 26.84 | 32.46 | 40.24 | 77.88 | 83.28 | 77.42 | 80.92 |
| 180 | 0.15 | 100 | 23.49 | 29.66 | 18.65 | 34.64 | 37.74 | 72.06 | 74.65 | 76.10 | 78.81 |
| 240 | 0.15 | 100 | 18.63 | 23.15 | 7.31 | 40.73 | 29.97 | 67.29 | 71.58 | 73.61 | 80.54 |
| 300 | 0.17 | 100 | 38.48 | 38.16 | 33.55 | 38.36 | 39.69 | 64.45 | 65.53 | 75.78 | 75.95 |
| Average | 0.13 | 100 | 28.36 | 26.37 | 22.23 | 34.91 | 40.55 | 71.75 | 73.08 | 76.82 | 80.23 |
|  | | | | | | | | | | | |
| (*Z*)-2-Alkenals | | | | | | | | | | | |
| 0 (Unheated) | - | - | - | - | - | - | - | - | - | - | - |
| 60 | 0.05 | 100 | 16.89 | 5.12 | 40.21 | 14.19 | 0.00 | sup | sup | sup | sup |
| 120 | 0.08 | 100 | 19.35 | 16.75 | 21.43 | 24.17 | 53.22 | sup | sup | sup | sup |
| 180 | 0.13 | 100 | 38.52 | 31.28 | 27.49 | 40.62 | 58.39 | 70.99 | sup | sup | sup |
| 240 | 0.16 | 100 | 16.22 | 12.95 | 8.25 | 39.23 | 48.28 | 77.56 | 80.28 | sup | 90.68 |
| 300 | 0.24 | 100 | 43.56 | 44.41 | 46.84 | 44.37 | 59.41 | 84.05 | 79.97 | 84.62 | 90.05 |
| Average | 0.11 | 100 | 26.91 | 22.10 | 28.84 | 32.51 | 43.86 | 77.54 | 80.13 | 84.62 | 90.37 |

Across the table, percentage suppression activity of LOPs by PDMS for each PDMS concentration is with respect to the LOPs concentration measured in the control (unstirred) sunflower oil thermally stressed at the corresponding thermo-oxidation duration (min). Not applicable (n/a) implies no measurement at the corresponding thermo-oxidation duration (min).

**Table S.1.** (*Continued*) Percentage suppression activity of LOPs by PDMS in PDMS-treated unstirred sunflower oil.

|  |  |  | Percentage suppression activity of LOPs by PDMS treatment | | | | | | | | |
| --- | --- | --- | --- | --- | --- | --- | --- | --- | --- | --- | --- |
| Thermo-oxidation duration (min) | Control (Unstirred) (mM) | Control (Unstirred) (% equivalence) | 6.25 × 10⁻⁷ ppm | 1.0 × 10⁻⁵ ppm | 0.025 ppm | 0.05 ppm | 0.1 ppm | 0.5 ppm | 1.0 ppm | 5.0 ppm | 10.0 ppm |
| Unidentified unsaturated aldehyde (signal k) | | | | | | | | | | | |
| 0 (Unheated) | - | - | - | - | - | - | - | - | - | - | - |
| 60 | 0.06 | 100 | sup | sup | sup | sup | sup | sup | sup | sup | sup |
| 120 | 0.07 | 100 | 3.62 | 18.02 | 30.51 | 30.66 | sup | sup | sup | sup | sup |
| 180 | 0.10 | 100 | 36.11 | 44.90 | 44.32 | 45.01 | 61.11 | sup | sup | sup | sup |
| 240 | 0.12 | 100 | 16.06 | 35.27 | 31.68 | 48.21 | 47.93 | sup | sup | sup | sup |
| 300 | 0.14 | 100 | 43.50 | 54.87 | 64.21 | 55.30 | 59.91 | sup | 68.40 | sup | sup |
| Average | 0.08 | 100 | 24.82 | 38.26 | 42.68 | 44.79 | 56.32 | sup | 68.40 | sup | sup |

Across the table, percentage suppression activity of LOPs by PDMS for each PDMS concentration is with respect to the LOPs concentration measured in the control (unstirred) sunflower oil thermally stressed at the corresponding thermo-oxidation duration (min). Not applicable (n/a) implies no measurement at the corresponding thermo-oxidation duration (min).

**Table S.2.** Percentage suppression activity of LOPs by PDMS in PDMS-treated stirred sunflower oil.

|  | Percentage suppression activity of LOPs by PDMS treatment | | | | | |
| --- | --- | --- | --- | --- | --- | --- |
| Thermo-oxidation duration (min) | Control (Stirred) (mM) | Control (Stirred) (% equivalence) | 0.5 ppm | 1.0 ppm | 5.0 ppm | 10.0 ppm |
| (*E*)-2-Alkenals | | | | | | |
| 0 (Unheated) | 0.16 | n/a | n/a | n/a | n/a | n/a |
| 60 | 1.57 | 100 | 63.56 | 73.06 | 70.97 | 70.09 |
| 120 | 2.96 | 100 | 70.01 | 79.46 | 83.56 | 83.36 |
| 180 | 3.59 | 100 | 63.73 | 76.08 | 76.34 | 81.23 |
| 240 | 4.99 | 100 | 63.69 | 78.13 | 74.76 | 78.92 |
| 300 | 5.36 | 100 | 58.32 | 68.82 | 69.62 | 70.86 |
| Average | 3.10 | 100 | 63.86 | 75.11 | 75.05 | 76.89 |
|  | | | | | | |
| (*E*,*E*)-2,4-Alkadienals | | | | | | |
| 0 (Unheated) | 0.04 | n/a | n/a | n/a | n/a | n/a |
| 60 | 1.47 | 100 | 64.88 | 75.69 | 73.42 | 78.93 |
| 120 | 1.98 | 100 | 61.15 | 73.23 | 79.14 | 78.17 |
| 180 | 2.30 | 100 | 54.59 | 68.66 | 69.55 | 75.11 |
| 240 | 2.82 | 100 | 53.28 | 69.57 | 65.08 | 70.03 |
| 300 | 2.77 | 100 | 45.72 | 57.29 | 56.21 | 58.86 |
| Average | 1.90 | 100 | 55.92 | 68.89 | 68.68 | 72.22 |
|  | | | | | | |
| 4,5-Epoxy-(*E*)-alkenals | | | | | | |
| 0 (Unheated) | - | - | - | - | - | - |
| 60 | 0.24 | 100 | 68.18 | 75.03 | 71.02 | 82.74 |
| 120 | 0.40 | 100 | 72.16 | 79.10 | 85.18 | 82.20 |
| 180 | 0.46 | 100 | 66.30 | 76.05 | 78.62 | 81.92 |
| 240 | 0.62 | 100 | 66.09 | 77.89 | 75.88 | 80.04 |
| 300 | 0.63 | 100 | 59.97 | 69.23 | 69.25 | 72.14 |
| Average | 0.39 | 100 | 66.54 | 75.46 | 75.99 | 79.81 |

Across the table, percentage suppression activity of LOPs by PDMS for each PDMS concentration is with respect to the LOPs concentration measured in the control (unstirred) sunflower oil thermally stressed at the corresponding thermo-oxidation duration (min). Not applicable (n/a) implies no measurement at the corresponding thermo-oxidation duration (min).

**Table S.2.** (*Continued*) Percentage suppression activity of LOPs by PDMS in PDMS-treated stirred sunflower oil.

|  | Percentage suppression activity of LOPs by PDMS treatment | | | | | |
| --- | --- | --- | --- | --- | --- | --- |
| Thermo-oxidation duration (min) | Control (Stirred) (mM) | Control (Stirred) (% equivalence) | 0.5 ppm | 1.0 ppm | 5.0 ppm | 10.0 ppm |
| 4-Hydroxy-(*E*)-2-alkenals | | | | | | |
| 0 (Unheated) | - | - | - | - | - | - |
| 60 | 0.30 | 100 | 66.55 | 73.36 | 70.72 | 79.77 |
| 120 | 0.56 | 100 | 72.54 | 80.80 | 84.71 | 84.79 |
| 180 | 0.63 | 100 | 65.07 | 76.07 | 76.95 | 82.06 |
| 240 | 0.80 | 100 | 62.52 | 76.05 | 73.26 | 78.17 |
| 300 | 0.79 | 100 | 52.84 | 63.76 | 64.37 | 67.30 |
| Average | 0.51 | 100 | 63.90 | 74.01 | 74.00 | 78.42 |
|  | | | | | | |
| 4-Hydroperoxy-(*E*)-2-alkenals | | | | | | |
| 0 (Unheated) | - | - | - | - | - | - |
| 60 | 0.22 | 100 | 64.05 | 73.43 | 73.33 | 77.39 |
| 120 | 0.39 | 100 | 69.17 | 79.51 | 83.62 | 82.69 |
| 180 | 0.44 | 100 | 61.66 | 75.72 | 76.51 | 81.88 |
| 240 | 0.59 | 100 | 64.08 | 76.81 | 75.09 | 79.33 |
| 300 | 0.59 | 100 | 55.91 | 66.85 | 66.96 | 70.99 |
| Average | 0.37 | 100 | 62.97 | 74.46 | 75.10 | 78.46 |
|  | | | | | | |
| (*Z*,*E*)-2,4-Alkadienals | | | | | | |
| 0 (Unheated) | 0.07 | 100 | n/a | n/a | n/a | n/a |
| 60 | 0.41 | 100 | 64.00 | 73.55 | 69.37 | 76.80 |
| 120 | 0.57 | 100 | 64.64 | 73.40 | 79.99 | 76.17 |
| 180 | 0.56 | 100 | 55.16 | 66.81 | 66.82 | 70.43 |
| 240 | 0.64 | 100 | 50.50 | 65.32 | 60.38 | 66.04 |
| 300 | 0.57 | 100 | 39.99 | 47.23 | 48.42 | 48.49 |
| Average | 0.47 | 100 | 54.86 | 65.26 | 65.00 | 67.59 |

Across the table, percentage suppression activity of LOPs by PDMS for each PDMS concentration is with respect to the LOPs concentration measured in the control (unstirred) sunflower oil thermally stressed at the corresponding thermo-oxidation duration (min). Not applicable (n/a) implies no measurement at the corresponding thermo-oxidation duration (min).

**Table S.2.** (*Continued*) Percentage suppression activity of LOPs by PDMS in PDMS-treated stirred sunflower oil.

|  | Percentage suppression activity of LOPs by PDMS treatment | | | | | |
| --- | --- | --- | --- | --- | --- | --- |
| Thermo-oxidation duration (min) | Control (Stirred) (mM) | Control (Stirred) (% equivalence) | 0.5 ppm | 1.0 ppm | 5.0 ppm | 10.0 ppm |
| *n*-Alkanals | | | | | | |
| 0 (Unheated) | 0.17 | 100 | n/a | n/a | n/a | n/a |
| 60 | 1.03 | 100 | 65.17 | 73.60 | 72.46 | 83.55 |
| 120 | 1.76 | 100 | 70.41 | 79.21 | 84.46 | 83.32 |
| 180 | 2.03 | 100 | 63.45 | 76.99 | 77.40 | 81.82 |
| 240 | 2.64 | 100 | 59.59 | 77.27 | 74.90 | 79.05 |
| 300 | 2.74 | 100 | 54.65 | 74.06 | 68.18 | 69.55 |
| Average | 1.73 | 100 | 62.65 | 76.23 | 75.48 | 79.46 |
|  | | | | | | |
| 4-Oxo-alkanals | | | | | | |
| 0 (Unheated) | - | - | - | - | - | - |
| 60 | 0.08 | 100 | 50.89 | 63.07 | 58.75 | 78.22 |
| 120 | 0.19 | 100 | 67.92 | 75.00 | 83.58 | 84.80 |
| 180 | 0.27 | 100 | 64.40 | 72.66 | 78.69 | 83.85 |
| 240 | 0.36 | 100 | 59.94 | 73.71 | 74.95 | 78.09 |
| 300 | 0.40 | 100 | 55.66 | 63.64 | 67.17 | 67.89 |
| Average | 0.22 | 100 | 59.76 | 69.62 | 72.63 | 78.57 |
|  | | | | | | |
| *n*-Alkanals (low molecular weight) | | | | | | |
| 0 (Unheated) | - | - | - | - | - | - |
| 60 | 0.15 | 100 | 64.80 | 76.08 | 71.05 | 86.98 |
| 120 | 0.19 | 100 | 61.94 | 71.73 | 81.23 | 79.11 |
| 180 | 0.19 | 100 | 49.77 | 64.90 | 69.29 | 72.99 |
| 240 | 0.20 | 100 | 39.20 | 63.78 | 62.33 | 64.53 |
| 300 | 0.17 | 100 | 27.23 | 34.06 | 37.76 | 44.64 |
| Average | 0.15 | 100 | 48.59 | 62.11 | 64.33 | 69.65 |

Across the table, percentage suppression activity of LOPs by PDMS for each PDMS concentration is with respect to the LOPs concentration measured in the control (unstirred) sunflower oil thermally stressed at the corresponding thermo-oxidation duration (min). Not applicable (n/a) implies no measurement at the corresponding thermo-oxidation duration (min).

**Table S.2.** (*Continued*) Percentage suppression activity of LOPs by PDMS in PDMS-treated stirred sunflower oil.

|  | Percentage suppression activity of LOPs by PDMS treatment | | | | | |
| --- | --- | --- | --- | --- | --- | --- |
| Thermo-oxidation duration (min) | Control (Stirred) (mM) | Control (Stirred) (% equivalence) | 0.5 ppm | 1.0 ppm | 5.0 ppm | 10.0 ppm |
| (*Z*)-2-Alkenals | | | | | | |
| 0 (Unheated) | 0.17 | 100 | n/a | n/a | n/a | n/a |
| 60 | 1.03 | 100 | 65.17 | 73.60 | 72.46 | 83.55 |
| 120 | 1.76 | 100 | 70.41 | 79.21 | 84.46 | 83.32 |
| 180 | 2.03 | 100 | 63.45 | 76.99 | 77.40 | 81.82 |
| 240 | 2.64 | 100 | 59.59 | 77.27 | 74.90 | 79.05 |
| 300 | 2.74 | 100 | 54.65 | 74.06 | 68.18 | 69.55 |
| Average | 1.73 | 100 | 62.65 | 76.23 | 75.48 | 79.46 |
|  | | | | | | |
| Unidentified unsaturated aldehyde (signal k) | | | | | | |
| 0 (Unheated) | - | - | - | - | - | - |
| 60 | 0.08 | 100 | sup | sup | sup | sup |
| 120 | 0.09 | 100 | sup | sup | sup | sup |
| 180 | 0.09 | 100 | sup | sup | sup | sup |
| 240 | 0.12 | 100 | sup | sup | sup | sup |
| 300 | 0.08 | 100 | sup | sup | sup | sup |
| Average | 0.08 | 100 | sup | sup | sup | sup |

Across the table, percentage suppression activity of LOPs by PDMS for each PDMS concentration is with respect to the LOPs concentration measured in the control (unstirred) sunflower oil thermally stressed at the corresponding thermo-oxidation duration (min). Not applicable (n/a) implies no measurement at the corresponding thermo-oxidation duration (min).
